# A Systematic Review Evaluating Psychometric Properties of Parent or Caregiver Report Instruments on Child Maltreatment: Part 2: Internal Consistency, Reliability, Measurement Error, Structural Validity, Hypothesis Testing, Cross-Cultural Validity, and Criterion Validity

**DOI:** 10.1177/1524838020915591

**Published:** 2020-04-09

**Authors:** Sangwon Yoon, Renée Speyer, Reinie Cordier, Pirjo Aunio, Airi Hakkarainen

**Affiliations:** 1 Department of Special Needs Education, Faculty of Educational Sciences, University of Oslo, Norway; 2 School of Occupational Therapy, Social Work and Speech Pathology, Faculty of Health Sciences, Curtin University, Perth, Australia; 3 Department of Otorhinolaryngology and Head and Neck Surgery, Leiden University Medical Centre, the Netherlands; 4 Department of Social Work, Education and Community Wellbeing, Faculty of Health and Life Sciences, Northumbria University, Newcastle, United Kingdom; 5 Department of Education, University of Helsinki, Finland; 6 Open University, University of Helsinki, Finland

**Keywords:** assessment, caregiver-reported measures, child abuse, child neglect, COSMIN, measurement properties, parent-reported measures

## Abstract

**Aims:**

Child maltreatment (CM) is global public health issue with devastating lifelong consequences. Global organizations have endeavored to eliminate CM; however, there is lack of consensus on what instruments are most suitable for the investigation and prevention of CM. This systematic review aimed to appraise the psychometric properties (other than content validity) of all current parent- or caregiver-reported CM instruments and recommend the most suitable for use.

**Method:**

A systematic search of the CINAHL, Embase, ERIC, PsycINFO, PubMed, and Sociological Abstracts databases was performed. The evaluation of psychometric properties was conducted according to the COnsensus-based Standards for the selection of health Measurement INstruments (COSMIN) guidelines for systematic reviews of patient-report outcome measures. Responsiveness was beyond the scope of this systematic review, and content validity has been reported on in a companion paper (Part 1). Only instruments developed and published in English were included.

**Results:**

Twenty-five studies reported on selected psychometric properties of 15 identified instruments. The methodological quality of the studies was overall adequate. The psychometric properties of the instruments were generally indeterminate or not reported due to incomplete or missing psychometric data; high-quality evidence on the psychometric properties was limited.

**Conclusions:**

No instruments could be recommended as most suitable for use in clinic and research. Nine instruments were identified as promising based on current psychometric data but would need further psychometric evidence for them to be recommended.

Child maltreatment (CM) is a major public health issue. More than half of the world’s children (1 billion children aged 2–17 years) are exposed to CM (Hillis et al., 2016). Approximately 155,000 children younger than 15 years die worldwide annually as a result of CM (Gilbert et al., 2009), which is the second leading cause of childhood death (Johnson, 2002). Furthermore, early exposure to CM has resulted in short-term and long-term devastating consequences from childhood to adulthood, such as behavioral problems, poor academic performance in childhood (Boden et al., 2007; Godinet et al., 2014), mental health problems, and experiencing poverty in adulthood (Currie & Spatz Widom, 2010; Kisely et al., 2018; Sugaya et al., 2012).

Due to the worldwide high prevalence and serious consequences of CM, the United Nations (UN) and World Health Organization (WHO) have urged that member states not only enact laws for the abolition of CM but also take action to investigate and prevent CM in each country (Hillis et al., 2016). In 1989, the UN (1989) presented the Convention on the Rights of the Child to protect children against all forms of abuse and neglect; the Convention was ratified by 196 member nations. Ten years later, the WHO (1999) published the Report of the Consultation on Child Abuse Prevention to provide global guidelines for investigation and prevention of CM based on international expert consensus. Recently, the UN (2015) has launched a new commitment to end CM as part of their 2030 Agenda for Sustainable Development Goals; all member states will evaluate their progress from 2016 to 2030 toward this goal for elimination of CM.

The task of monitoring progress toward elimination of CM is complicated by the trend that the prevalence of CM tends to underestimate the true incidence because information about the CM prevalence mostly relies on professional reports (from child protection workers, doctors, and teachers, who are mandated to report CM) rather than parent/carer or child reports (Shanahan et al., 2018). As CM usually occurs in private places, such as homes, in the absence of witnesses and is mostly perpetrated by parents (Institute of Medicine and National Research Council, 2014), actual incidences of CM are difficult to be accurately reported by individuals other than parents/carers or children. For this reason, parent/carer or child reports are the only way to determine the true incidence of CM that is committed, instead of relying on professional reports (Miller-Perrin & Perrin, 2013).

A recent meta-analysis on the prevalence of caregiver-perpetrated CM has shown that prevalence rates based on child reports is far lower than when based on caregiver reports (Devries et al., 2018) due to recall bias (i.e., difficulty remembering past events; Greenhoot, 2011; Milner & Crouch, 1997). In addition, even though caregiver reports on their own perpetration of CM appear not to underestimate, the accuracy of caregiver reports is still a subject for debate due to social desirability bias (i.e., the tendency to respond in a socially desirable way; Della Femina et al., 1990; Milner & Crouch, 1997). Thus, identifying high-quality parent or caregiver report instruments is essential to accurately estimate prevalence of CM.

The choice of high-quality instruments is strongly determined by having robust psychometric properties such as validity and reliability (Karanicolas et al., 2009). The best way to select the most reliable and valid instruments is to systematically review the literature on its psychometric properties (Scholtes et al., 2011). Good systematic reviews of psychometric properties of instruments should evaluate the quality of the studies on psychometric properties of an instrument, evaluate the quality of psychometric properties of an instrument, and synthesize the findings from all the psychometric studies using consensus-based standards and methods (Terwee et al., 2016). Recently, the COnsensus-based Standards for the selection of health Measurement INstruments (COSMIN) group has published guidelines for conducting systematic reviews on psychometric properties of patient-reported outcome instruments (Prinsen et al., 2018; Terwee et al., 2018). The COSMIN guidelines include the following practical tools: a taxonomy defining each psychometric property (Mokkink et al., 2010b), a checklist to assess methodological quality of psychometric studies (Mokkink, de Vet et al., 2018), criteria to assess each result of single study on a psychometric property (Prinsen et al., 2018; Terwee et al., 2018), and a rating system summarizing all results of studies on each psychometric property and grading quality of all evidence used for the assessments of both the methodological and the psychometric quality (Prinsen et al., 2018; Terwee et al., 2018).

The COSMIN taxonomy provides consensus-based terminology and definitions on nine psychometric properties, which forms the following three domains (Mokkink et al., 2010b): (1) validity (the extent to which an instrument measures the construct it is intended to measure), (2) reliability (the extent to which scores for patients who have not changed are the same for repeated measurements), and (3) responsiveness (the ability to detect clinically important change over time in the construct measured). The following psychometric properties are part of the validity domain (Mokkink et al., 2010b): (1) content validity (extent to which the content of an instrument adequately reflects the construct measured), (2) criterion validity (extent to which the scores adequately reflect a gold standard), and (3) construct validity (extent to which the scores are consistent with hypotheses based on the assumption that an instrument validly measures the construct measured). Construct validity is subdivided into the following three psychometric properties: (3.1) structural validity (extent to which the scores adequately reflect the dimensionality of the construct measured), (3.2) hypothesis testing (extent to which the scores are consistent with hypotheses on differences between relevant groups and relations to scores of other instruments), and (3.3) cross-cultural validity (extent to which a translated or culturally adapted version of an instrument adequately reflects the performance of the items of the original instrument). The following three psychometric properties comprise the reliability domain (Mokkink et al., 2010b): internal consistency (degree of the interrelatedness of items), reliability (the proportion of total score variance which is due to true differences among respondents), and measurement error (systematic and random error of a respondent’s score that is not due to true changes in the construct being measured). Responsiveness is a separate domain (Mokkink et al., 2010b).

The most significant advantage of the COSMIN guidelines over other methods is that they were designed to assess the quality of *all* domains of psychometric properties comprehensively, while other methods were designed for evaluating limited aspects of psychometric properties only. For example, the revised Quality Assessment of Diagnostic Accuracy Studies (QUADAS-2) checklist (Whiting et al., 2011) mainly focuses on the single measurement property of criterion validity (Christian et al., 2019), whereas the Quality Appraisal of Reliability Studies (QAREL) checklist (Lucas et al., 2010) was designed for evaluating reliability only (Abedi et al., 2019). Furthermore, compared with the COSMIN guidelines, both the QUADAS-2 and QAREL checklists have more criteria that rely on subjective interpretation of psychometric reporting to determine the quality of psychometric studies (Abedi et al., 2019; Christian et al., 2019).

Another point of difference is that the COSMIN system deviates from earlier appraisal methods in that construct validity can be evaluated through hypothesis testing, structural validity, and cross-cultural validation. Hypothesis testing involves determining the presence and magnitude of relationships between items of instruments following the traditional multitrait-multimethod (MTMM) approach (Campbell & Fiske, 1959). In turn, structural validity should be evaluated by determining the relationships between the hypothesized and observed factor structure by conducting modern confirmatory factor analysis (CFA; Prinsen et al., 2018). According to the COSMIN guidelines, evidence on structural validity should be considered more important than hypothesis testing when recommending instruments in terms of construct validity (Prinsen et al., 2018), as CFA is a more robust approach than the MTMM in evaluating construct validity. The reasons are 2-fold: first, CFA is more accurate in determining measurement error than the MTMM (Gaither, 1993); and second, Campbell and Fiske’s method (1959) were based on a subjective interpretation of rules of thumb criteria of the MTMM correlations, which lacked clear standards to differentiate satisfactory and unacceptable results (Shen, 2017). An additional advantage of using the COSMIN guidelines is that both traditional (classic test theory) and contemporary psychometric theories (item response theory) can be employed to evaluate the quality of psychometric properties of an instrument (Prinsen et al., 2018). However, although the COSMIN guidelines are comprehensive, precise, and balanced, it is complex and requires in-depth knowledge of psychometrics and quality rating criteria for conducting systematic reviews of the psychometric properties of an instrument (Christian et al., 2019; Dobbs et al., 2019).

To date, two systematic reviews have evaluated the psychometric characteristics of CM instruments: Kim et al. (2016) and Saini et al. (2019). Kim et al. (2016) conducted a systematic review to evaluate the methodological quality of studies reporting on the development of CM instruments using the 14 criteria of the QUADAS (Whiting et al., 2003), which is an assessment tool for methodological quality of psychometric studies. However, the authors did not evaluate the psychometric quality of the included instruments. Another systematic review by Saini et al. (2019) evaluated both the study quality and psychometric quality of the CM instruments. However, the authors mainly identified and evaluated child self-report and clinician-report interview instruments, excluding parent- or caregiver-reported CM instruments. Moreover, the authors did not use the latest, thoroughly revised COSMIN guidelines (Prinsen et al., 2018; Terwee et al., 2018), but instead used a previous version of the COSMIN checklist (Mokkink et al., 2010a) and criteria (Terwee et al., 2007) for quality assessment of included studies and instruments. The previous version of checklist and criteria does not have specific and comprehensive standards for assessing content validity, even though it is the most important psychometric property, nor do the guidelines have a standardized method to synthesize psychometric data (Prinsen et al., 2018; Terwee et al., 2018). To overcome these weaknesses of the previous version, the COSMIN guidelines (Prinsen et al., 2018; Terwee et al., 2018) were completely revised in recent years. The COSMIN guidelines recommend evaluating content validity of an instrument first because if it is unclear what construct(s) the instrument is actually measuring, the evaluation of the other psychometric properties is meaningless (Mokkink, Prinsen, et al., 2018; Prinsen et al., 2018). In other words, if reviews find high-quality evidence that an instrument has insufficient content validity, the other psychometric properties of the instrument do not need to be further evaluated. Accordingly, the content validity of the parent- or caregiver-reported CM instruments was evaluated first in a companion paper (Part 1; Yoon et al., 2020). As no high-quality evidence of insufficient content validity was found, this present review (Part 2) continued to evaluate the other psychometric properties of the included parent- or caregiver-reported CM instruments. To date, no systematic review on the psychometric properties of parent- or caregiver-reported CM instruments has been published.

## Study Aim

The aim of this systematic review (Part 2) was to evaluate psychometric properties (other than content validity) of all current parent- or caregiver-reported CM instruments and to recommend the most suitable parent- or caregiver-reported CM instruments using the COSMIN guidelines (Prinsen et al., 2018). Content validity has been evaluated and reported on in a companion paper (Part 1; Yoon et al., 2020).

## Method

**Figure 1.**
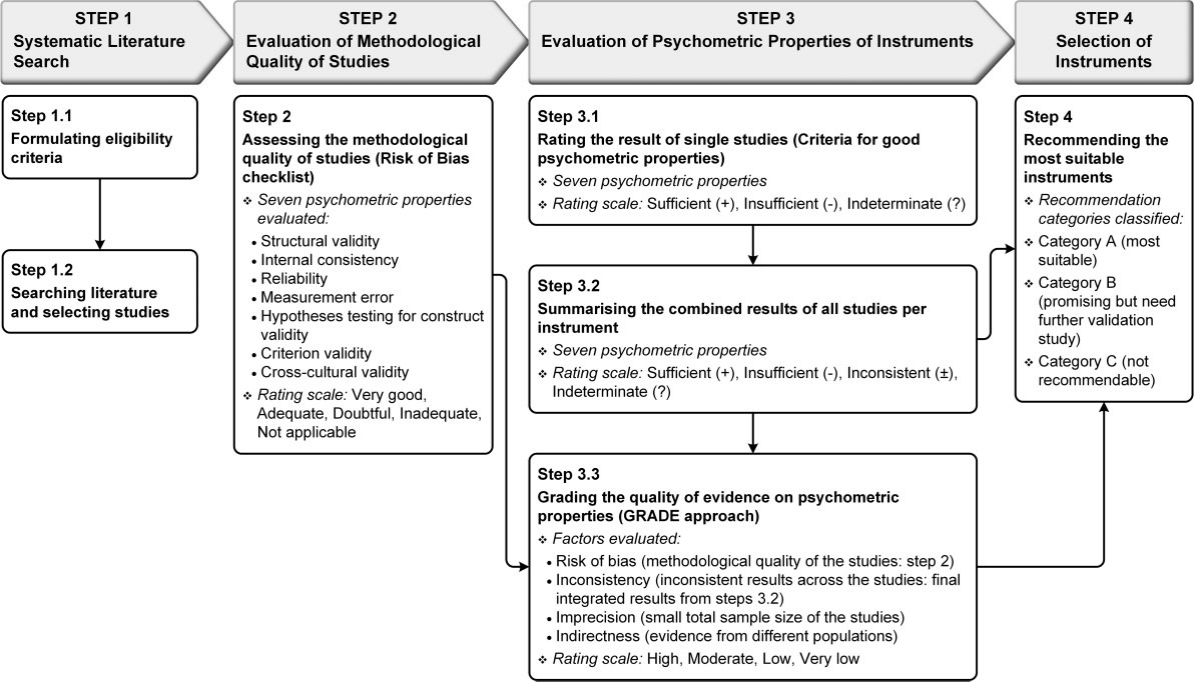
Study design: Steps for preferred reporting items for systematic reviews and meta-analyses and consensus-based standards for the selection of health measurement instruments processes. *Note*. Responsiveness was outside the scope of this review; Content validity was evaluated in a companion paper (Part 1; Yoon et al., 2020).

This systematic review followed the Preferred Reporting Items for Systematic reviews and Meta-Analyses (PRISMA) statement (Moher et al., 2009) and the COSMIN guidelines (Prinsen et al., 2018). This review was conducted in four sequential steps (see Figure 1):

- Step 1: *Systematic literature search* formulating eligibility criteria (Step 1.1) and searching the literature and selecting studies (Step 1.2);
- Step 2: *Evaluation of the methodological quality of studies* on psychometric properties of instruments using the COSMIN Risk of Bias checklist;
- Step 3: *Evaluation of the psychometric properties of instruments* rating the result of single studies against the criteria for good psychometric properties (Step 3.1), summarizing all results of studies per instrument (Step 3.2), and grading the quality of evidence on psychometric properties (Step 3.3); and
- Step 4: *Selection of instruments* recommending the most suitable instruments.

Each of these steps will be further described in the sections that follow.

### Step 1: Systematic Literature Search

Systematic literature search for this review was performed in two substeps: formulating eligibility criteria (Step 1.1) and searching literature and selecting studies (Step 1.2). These two steps are in agreement with the PRISMA statement (Moher et al., 2009).

#### Eligibility criteria (Step 1.1)

To be included for this review, instruments needed to meet the following four eligibility criteria: (1) parent or caregiver report instruments; (2) instruments were developed and published in English; (3) instruments assessed parents’ or caregivers’ attitude toward CM or perpetration of CM; (4) to ensure that an instrument reflects an overarching construct of CM, at least one subscale or a minimum of 30% of all items within an instrument measured one or more of the four main types of CM, including physical abuse (acts causing actual or potential physical harm to a child), emotional abuse (acts having adverse impact on the child’s emotional development), sexual abuse (acts using a child for sexual gratification), neglect (failure providing for the development of a child in health, education, emotional development, nutrition, shelter, and safe living conditions; Krug et al., 2002; WHO, 1999).

The following two additional selection criteria were used for psychometric studies: (1) Journal articles and manuals were published in English; (2) reported psychometric data of at least one of the following eight psychometric properties as defined in the COSMIN taxonomy (Mokkink et al., 2010b): structural validity, internal consistency, reliability, measurement error, hypotheses testing for construct validity, criterion validity, cross-cultural validity, and content validity. Responsiveness was beyond the scope of the present review, and content validity was assessed in a companion paper (Part 1; Yoon et al., 2020).

#### Literature search and study selection (Step 1.2)

Systematic literature searches were conducted in six electronic databases: CINAHL, Embase, ERIC, PsycINFO, PubMed, and Sociological Abstracts. All database searches were conducted in January 2018 with an updated search conducted in October 2019. Subject headings and free text words were used to search databases and to retrieve all journal articles up until October 2019 (see Supplementary Appendix A).

Abstracts identified by database searches were screened to retrieve eligible instruments and full-text articles on any psychometric property by two independent reviewers. One reviewer screened all abstracts while the other reviewer screened a randomly selection of half of all abstracts. All full texts of eligible abstracts were extracted and screened independently by two reviewers. Any differences between two reviewers were resolved through consensus with a third reviewer. The interrater agreement was assessed by calculating weighted κ (Cohen & Humphreys, 1968) and interpreted as very good (0.81–1.00), good (0.61–0.80), moderate (0.41–0.60), fair (0.21–0.40), and poor (0.00–0.20; Altman, 1991).

Next, reference lists of all included full texts were hand searched to identify additional eligible instruments and studies. Websites of two major publishers of measurements in social science (Pearson and Western Psychological Services) were also searched to identify potential instruments and manuals. Both searches for reference lists and websites were conducted by one reviewer and the identified additional instruments and studies were checked by the other reviewer. When instruments were not published or available for free, the developers of the instruments were contacted to obtain the original instruments.

### Step 2: Evaluation of Methodological Quality of Studies

The methodological quality of the studies on the psychometric properties of the included instruments was rated using the COSMIN Risk of Bias checklist (Mokkink, de Vet et al., 2018), which is a standardized tool for evaluating study quality of psychometric studies. The checklist contains 3–38 items for each psychometric property (Mokkink, de Vet et al., 2018). The checklist items rate the quality of study design and the robustness of statistical analyses conducted in studies on any of the seven psychometric properties evaluated in this article (Mokkink, de Vet et al., 2018). Evaluation of reliability included all three aspects (Mokkink et al., 2010b): test–retest reliability (the degree of total score variance in repeated measurement on the same patients over time), interrater reliability (the degree of total score variance in repeated measurement on the same occasions by different raters), and intrarater reliability (the degree of total score variance in repeated measurement on different occasions by the same rater). Cross-cultural validity was evaluated for measurement invariance of an instrument across culturally different groups (e.g., nationality, gender, and age) within English-speaking populations only (Mokkink, de Vet et al., 2018), due to including only instruments developed and published in English in this review. Furthermore, evaluation of criterion validity involved exploring associations between an instrument and a gold standard, as well as between an original long version and the shortened version thereof (Mokkink, Prinsen, et al., 2018). Lastly, hypothesis testing for construct validity was evaluated by appraising the associations between two instruments to determine whether they are measuring a similar construct of interest (i.e., convergent validity) and to compare differences in scores between subgroups of the target population (i.e., discriminative validity; Mokkink, de Vet et al., 2018).

When rating the methodological quality of the included studies on psychometric properties, each checklist item was ranked on a 4-point rating scale: 1 = *inadequate*, 2 = *doubtful*, 3 = *adequate*, and 4 = *very good* (Mokkink, de Vet et al., 2018). A total rating for each psychometric property was obtained by calculating the ratio between “the obtained total score minus the minimum score possible’ and ‘the maximum score possible minus the minimum score possible” (Cordier et al., 2015). This approach was adopted instead of a worst score counts method (i.e., reporting total ratings obtained by taking the lowest rating among any of the checklist items) recommended by COSMIN guideline (Mokkink, Prinsen, et al., 2018), as determining the total ratings entirely based on the lowest rating single item tends to impede the detection of subtle differences in methodological quality between studies (Speyer et al., 2014). Therefore, the total score of methodological quality ratings per psychometric property was presented as a percentage of the ratings: inadequate (0%–25%), doubtful (25.1%–50%), adequate (50.1%–75%), and very good (75.1%–100%). Two reviewers rated the methodological quality independently, and any discrepancies were resolved by consensus. The interrater agreement between two reviewers was determined by calculating the weighted κ (Cohen & Humphreys, 1968).

After evaluating methodological quality of the included psychometric studies, the following data were extracted from the included studies and instruments (Mokkink, Prinsen, et al., 2018): (1) study characteristics (i.e., study purpose, assessed psychometric properties, and study population); (2) instrument characteristics (i.e., instrument names, construct to be measured, target population, purpose of use, number of [sub] scales and items, and response options and recall period); and (3) study results on seven psychometric properties (internal consistency, reliability, measurement error, structural validity, hypothesis testing, cross-cultural validity, and criterion validity). One reviewer extracted all relevant data from included studies, and the other reviewer checked the extracted data for accuracy and completeness.

### Step 3: Evaluation of Psychometric Properties of Instruments

The psychometric properties of instruments were assessed for each of seven psychometric properties in three consecutive steps: Step 3.1 rating the result of single studies, Step 3.2 summarizing the results of all studies per instrument, and Step 3.3 grading the quality of evidence on psychometric properties. All ratings were conducted by two reviewers independently where after consensus ratings were determined by discussion between reviewers.

#### Rating the result of single studies (Step 3.1)

Rating the results of single studies was conducted for each psychometric property separately. The results of each psychometric property in each individual study were rated as sufficient (above the quality criteria threshold: +), insufficient (below the quality criteria threshold: −), or indeterminate (less robust data that do not meet the quality criteria:?), using the predefined criteria for good psychometric properties (Mokkink, Prinsen, et al., 2018; see Supplementary Appendix B).

#### Summarizing the results of all studies per instrument (Step 3.2)

All results on each psychometric property from available studies per instrument were qualitatively summarized into overall ratings of the psychometric property per instrument (Prinsen et al., 2018). An overall sufficient (+), insufficient (−) inconsistent (±), or indeterminate (?) rating was given for each psychometric property per instrument, with a 75% agreement rule used (Mokkink, Prinsen, et al., 2018): that is, for an overall sufficient (+) or insufficient (−) rating on a psychometric property, 75% or more of the studies reporting the psychometric property must be sufficient (+) or insufficient (−); otherwise, for an overall inconsistent (±) rating, less than 75% of studies showed the same rating; and for overall indeterminate (?) rating, all studies must be indeterminate (?).

#### Grading the quality of evidence on psychometric properties (Step 3.3)

The quality of the evidence (i.e., the total body of evidence used for overall ratings on each psychometric property of an instrument) was graded as high, moderate, low, or very low using a modified Grading of Recommendations Assessment, Development and Evaluation (GRADE) approach (Prinsen et al., 2018; see Supplementary Appendix C). The GRADE approach considers the initial quality of evidence used for overall ratings to be high, but the evidence quality is subsequently downgraded by one or more levels (to moderate, low, or very low) if there are serious (one level down: −1), very serious (two levels down: −2), or extremely serious (three levels down: −3) concerns. The following four factors were considered in determining the ratings: (a) risk of bias (limitations in the methodological quality of studies: Step 2), (b) inconsistency (unexplained heterogeneity in results of studies: Step 3.2), (c) indirectness (evidence from different populations than the targeted population in the review), and (d) imprecision (a low total number of samples included in the studies; Mokkink, Prinsen, et al., 2018). For example, for downgrading one level (from *high* to *moderate*), only one factor is allowed to have a serious concern (−1); for two levels (from *high* to *low*), either only one factor with a very serious concern (−2) or two factors with serious concerns (−1) is allowed; for three levels (from *high* to *very low*), one factor with an extremely serious concern (−3), one factor with very serious concern (−2), and one factor with serious (−1) to extremely serious concerns (−3), or more than three factors with serious (−1) to extremely serious concerns (−3) is allowed. Quality of evidence was not graded when the overall rating was indeterminate (?) as this indicates lack of robust evidence (Prinsen et al., 2018). Further details on grading quality of evidence can be found in the COSMIN usual manual for systematic reviews of instruments (Mokkink, Prinsen, et al., 2018).

### Step 4: Selection of Instruments

The selection of instruments and recommendation of suitable instruments for future use was based on combining overall rating results of each psychometric property (Step 3.2) and grading results of evidence quality for each property (Step 3.3; Prinsen et al., 2018). The recommendation was based on both findings of content validity (Part 1) and other psychometric properties (Part 2) of included instruments. Each instrument was classified into three recommendation categories (Mokkink, Prinsen, et al., 2018): (A) most suitable (i.e., instruments with high-quality evidence for sufficient content validity—in any aspects of relevance, comprehensiveness, and comprehensibility—and at least low-quality evidence for sufficient internal consistency); (B) promising but need further validation studies (i.e., instruments categorized not in A or C); and (C) not recommendable (i.e., instruments with high-quality evidence for an insufficient psychometric property).

To determine suitable instruments, content validity and internal consistency were considered as decisive psychometric properties rather than other properties because if it is unclear what an instrument is actually measuring and how different items in the instrument are related with construct to be measured, the evaluation of the other psychometric properties is meaningless. Furthermore, this review did not consider interpretability (the degree to which clinical meaning can be assigned to an instrument’s quantitative scores or change in scores) and feasibility (ease of use such as length, completion time, and access fee of an instrument) to recommend the most suitable CM instruments because neither interpretability nor feasibility is considered psychometric properties (Prinsen et al., 2018).

## Results

### Systematic Literature Search

**Figure 2.**
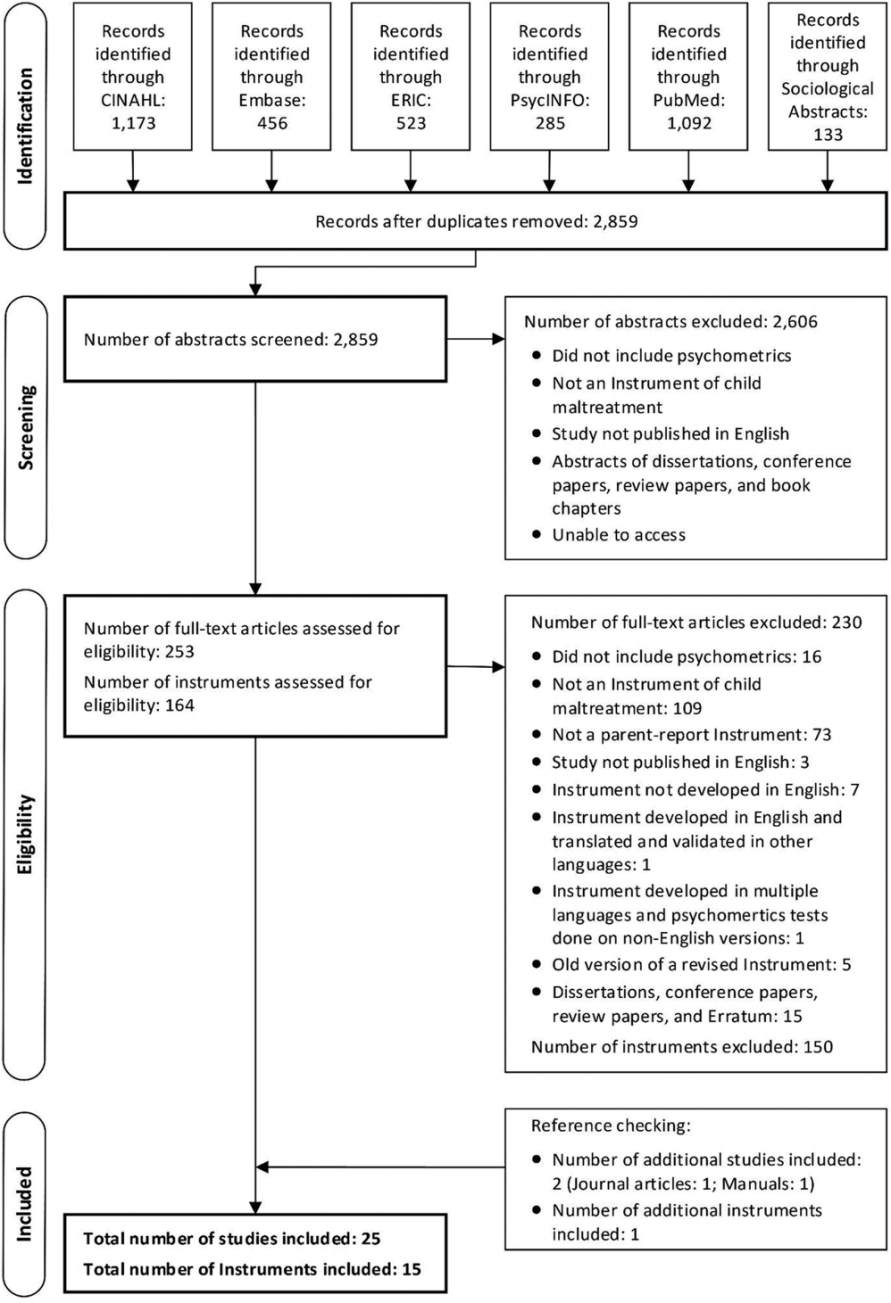
Flow diagram of the reviewing procedure based on Preferred Reporting Items for Systematic reviews and Meta-Analyses (Moher et al., 2009).

A total of 2,859 abstracts (removing duplicates) were retrieved from six databases: 1,173 records from CINAHL; 456 records from Embase; 523 records from ERIC; 285 records from PsycINFO; 1,092 records from PubMed; and 133 records from Sociological Abstracts. Figure 2 presents the flow chart of the studies and instruments identified during the searching literature and selecting studies (Step 1.2) according to the PRISMA (Moher et al., 2009). In total, 253 full-text articles and 164 instruments were assessed for eligibility, of which 23 articles and 14 instruments met all inclusion criteria: a list of the 150 excluded instruments and reasons for exclusion are provided in Supplementary Appendix D. Reference checking of the included 23 full-text articles identified two additional studies (one article and one manual) and one additional instrument met all inclusion criteria. As a result, 25 studies reporting and analyzing psychometric properties of 15 parent or carer report CM instruments were included in this review. The interreviewer agreement for study selection between two reviewers was very good (Altman, 1991): weighted κ for abstract selection  =  0.87 (95% confidence interval [CI] = [0.83, 0.90]); weighted κ for article selection = 0.86 (95% CI [0.77, 0.94]).

### Characteristics of Included Studies and Instruments

General characteristics of the psychometric studies of included CM instruments are presented in Supplementary Appendix E. Table 1 summarizes the characteristics of the included 15 instruments. All but three instruments were multidimensional, having some subscales to measure a range of different facets of CM, while the remaining instruments were a unidimensional scale. The majority of the instruments (14/15) were designed for current parent or carer respondents, except one instrument that was designed for prospective parents (i.e., before or during pregnancy) to reduce the risk of future CM. Ten instruments had a purpose of use for identifying maltreating parents/carers and/or evaluating intervention programs; four instruments for evaluating intervention programs; and one for identifying abused children by parents/carers.

**Table 1.** Characteristics of the Included Instruments for the Assessment of Child Maltreatment.

Instrument (References)	Construct	(Sub)scales	Target Population	Purpose of Use	Number of Items	Range of Score	Response Options	Recall Period
AAPI-2 (Bavolek & Keene, 1999; Conners et al., 2006; Lawson et al., 2017; Rodriguez et al., 2011; Russa & Rodriguez, 2010)	Abusive and neglecting parenting practices	Five (sub)scales: Inappropriate parental expectations; Parental lack of an empathic awareness of children’s needs; Strong belief in the use and value of corporal punishment; Parent child role reversal; Oppressing children’s power and independence	Current and prospective parent populations	Identification of maltreating parents/carers; Evaluation of intervention	40	0–50 (Raw total scores per subscale are converted into standard scores: range 0–10)	5-point ordinal scale (*strongly disagree* = 1 to *strongly disagree* = 5)	Not specified
APT (Rodriguez et al., 2011; Russa & Rodriguez, 2010)	Attitude toward physical discipline	Two (sub)scales: Physical discipline; Escalation of physical discipline	Prospective parent populations	Identification of maltreating parents/ carers	26	0–26	10 nominal scale (from nonphysical discipline tactics to physical discipline tactics)	Not specified
CNQ (Stewart et al., 2015)	Child neglect	Four (sub)scales: Physical neglect; Emotional neglect; Educational neglect; Supervision neglect	Parents with older children	Identification of maltreating parents/carers	46	46–184	4-point ordinal scale (*always* = 1 to *never* = 4)	Past 6 months
CNS-MMS (Kirisci et al., 2001)	Child neglect	One (sub)scales: Child neglect	Mothers	Evaluation of intervention	11	11–33	3-point ordinal scale (*hardly ever* = 1 to *often* = 3)	Past 6 months
CTS-ES (Lang & Connell, 2017)	Potentially traumatic event (including childhood physical abuse, sexual abuse, and domestic or community violence)	One (sub)scale: Potentially traumatic event	Caregivers	Identification of children maltreated by parents/carers	4	0–4	Dichotomous scale (*no* = 0 or *yes* = 1)	Not specified
CTSPC (Compier-de Block et al., 2017; Grasso et al., 2016; Kobulsky et al., 2017; Lorber & Slep, 2017; O’Dor et al., 2017; Rodriguez, 2010; Straus et al., 1998)	Physical and psychological child abuse	Three (sub)scales: Nonviolent discipline; Psychological aggression; Physical assault	Parents	Identification of maltreating parents/carers; Evaluation of intervention	22	0–550 (raw scores per item are converted into frequency scores: 0 = 0, 1 = 1, 2 = 2, 3–5 = 4, 6–10 = 8, 11–20 = 15, and > 20 = 25)	8-point ordinal scale (0 = *never happened*; 1 = o*nce in the past year*; 2 = *twice*; 3 = *3–5 times*; 4 = *6–10 times*; 5 = *11–20 times*; 6 = *more than 20 times*; 7 = *not in the past year, but it happened before*)	Past 1 year
FM-CA (Heyman et al., 2019)	Clinically significant child abuse and neglect	Two (sub)scales: Physical child abuse; Psychological child abuse	Parents	Identification of maltreating parents/carers; Evaluation of intervention	27	0–63	Dichotomous scale for physical child abuse subscale (*I did* = 0 or *I never did* = 1); 6-point ordinal scale for psychological child abuse subscale (*never* = 0 to *more than once a day* = 5)	Past 1 year
ICAST-Trial (Meinck et al., 2018)	Child abuse and neglect	Four (sub)scales: Physical abuse; Emotional abuse; Contact sexual abuse; Neglect	Caregivers	Evaluation of intervention	14	0–112	9-point ordinal scale (*never* = 0 to *more than 8 times* = 8)	Past 1 month
IPPS (Gordon et al., 1979)	Intensity of parent behavioral responses to hypothetical child misbehavior situations	Five (sub)scales: School misbehavior; Disobedience after a recent reminder; Public disobedience; Crying; Destructiveness	Parents	Identification of maltreating parents/carers; Evaluation of intervention	33	33–231	7-point ordinal scale (*no reaction* = 1 to *very strong punishment* = 7)	Not specified
MCNS (Lounds et al., 2004)	Maternal neglectful behavior towards their children	Four (sub)scales: Emotional neglect; Cognitive neglect; Supervisory neglect; Physical needs neglect	Mothers	Identification of maltreating parents/carers	20	20–80	4-point ordinal scale (*strongly disagree* = 1 to *strongly agree* = 4)	Past 1 year
MCNS-SF (Lounds et al., 2004)	Maternal neglectful behavior towards their children	Two (sub)scales: Emotional neglect; Cognitive neglect; Supervisory neglect; Physical needs neglect	Mothers	Identification of maltreating parents/carers	8	4–32	4-point ordinal scale (*strongly disagree* = 1 to *strongly agree* = 4)	Past 1 year
P-CAAM (Rodriguez et al., 2011)	Acceptance of parent-child aggression	Two (sub)scales: Physical discipline; Physical abuse	Current and prospective parent populations	Evaluation of intervention	8 video clips: 90 sec each	0–NR	Clips builds towards “initial physical contact between caregiver and child”; Rater should identify that moment and stop video; Delay between actual physical contact and stop video = score (per video)	Not specified
POQ (Azar & Rohrbeck, 1986; Haskett et al., 2006; Mammen et al., 2003)	Parental expectations of child behavior	Six (sub)scales: Self-care; Family responsibility and care of siblings; Help and affection to parents; Leaving children alone; Proper behavior and feelings; Punishment	Parents	Identification of maltreating parents/carers	60	0–60	Dichotomous scale (*disagree* = 0 or *agree* = 1)	Not specified
PRCM (Vittrup et al., 2006)	Discipline techniques in response to children’s misbehaviors	One (sub)scale: Discipline techniques	Parents with young children	Identification of maltreating parents/carers; Evaluation of intervention	12	0–72	6-point ordinal scale (never = 0–9 ≥ times per week = 6)	Past one week
SBS-SV (Russell, 2010)	Shaken baby syndrome awareness	Three (sub)scales: Soothing techniques; Discipline techniques; Potential for injury	Parents and caregivers of young children	Evaluation of intervention	36	36–216	6-point ordinal scale (*strongly disagree* = 1 to *strongly agree* = 6)	Not specified

*Note.* AAPI-2 = Adult Adolescent Parenting Inventory–2; APT = Analog Parenting Task; CNQ = Child Neglect Questionnaire; CNS-MMS = Child Neglect Scales–Maternal Monitoring and Supervision Scale; CTS-ES = Child Trauma Screen–Exposure Score; CTSPC = Conflict Tactics Scales: Parent–Child version; FM-CA = Family Maltreatment–Child Abuse criteria; ICAST-Trial = ISPCAN (International Society for the Prevention of Child Abuse and Neglect) Child Abuse Screening Tool for use in Trials; IPPS = Intensity of Parental Punishment Scale; MCNS = Mother–Child Neglect Scale; MCNS-SF = Mother–Child Neglect Scale–Short Form; P-CAAM = Parent–Child Aggression Acceptability Movie task; POQ = Parent Opinion Questionnaire; PRCM = Parental Response to Child Misbehavior questionnaire; SBS-SV = Shaken Baby Syndrome Awareness Assessment–Short Version.

### Methodological Quality of the Included Studies

The methodological quality of the 25 included studies (24 articles and 1 manual) was assessed using the COSMIN Risk of Bias checklist (Mokkink, de Vet et al., 2018). Some studies measured more than one psychometric property and included more than one instrument: the studies were rated multiple times for each psychometric property and instrument, respectively. For all 29 studies (including four duplicates), an overview of all methodological quality ratings is displayed in Table 2. Most studies reported on hypotheses testing for construct validity (25/29) and internal consistency (21/29). Only a small number of studies included psychometric data on structural validity (10 studies), reliability (5 studies), cross-cultural validity (1 study), and criterion validity (1 study). No information was retrieved on measurement error in any study. The interreviewer agreement for quality assessment of included studies between both reviewers was very good: weighted κ = 0.86 (95% CI [0.83, 0.90]).

**Table 2.** Methodological Quality Assessment of Studies on Psychometric Properties of the Included Instruments.

		Psychometric Property: Methodological Quality per Study^a^					
Instrument	Reference	Structural Validity	Internal Consistency	Cross-Cultural Validity	Reliability	Criterion Validity	Hypotheses Testing
AAPI-2	Bavolek and Keene (1999)	Very good (88.9%)	Very good (100.0%)	NR	NR	NR	Adequate (55.6%)
	Conners et al. (2006)	Very good (100.0%)	Very good (77.8%)	NR	NR	NR	Very good (81.3%)
	Lawson et al. (2017)	Adequate (66.7%)	Very good (100.0%)	NR	NR	NR	Adequate (66.7%)
	Rodriguez et al. (2011)	NR	Adequate (66.7%)	NR	NR	NR	Very good (100.0%)
	Russa and Rodriguez (2010)	NR	NR	NR	NR	NR	Very good (100.0%)
APT	Rodriguez et al. (2011)	NR	Very good (100.0%)	NR	NR	NR	Very good (83.3%)
	Russa and Rodriguez (2010)	NR	Very good (77.8%)	NR	NR	NR	Very good (90.0%)
CNQ	Stewart et al. (2015)	Adequate (75.0%)	Doubtful (33.3%)	NR	NR	NR	Very good (91.2%)
CNS-MMS	Kirisci et al. (2001)	Very good (100.0%)	Very good (100%)	NR	NR	NR	Very good (100.0%)
CTS-ES	Lang and Connell (2017)	NR	NR	NR	NR	NR	Very good (91.7%)
CTSPC	Compier-de Block et al. (2017)	NR	Very good (88.9%)	NR	Very good (77.8%)	NR	Adequate (55.6%)
	Cotter et al. (2018)	Very good (77.8%)	Adequate (55.6%)	NR	NR	NR	Very good (83.3%)
	Grasso et al. (2016)	NR	Very good (100.0%)	NR	NR	NR	NR
	Kobulsky et al. (2017)	NR	NR	NR	Very good (100.0%)	NR	NR
	Lorber and Slep (2017)	Very good (100.0%)	Adequate (58.3%)	NR	NR	NR	NR
	O’Dor et al. (2017)	NR	Very good (100.0%)	NR	NR	NR	Very good (100.0%)
	Rodriguez (2010)	NR	NR	NR	NR	NR	Very good (91.7%)
	Straus et al. (1998)	NR	Adequate (66.7%)	NR	NR	NR	Adequate (66.7%)
FM-CA	Heyman et al. (2019)	NR	NR	NR	NR	NR	Doubtful (41.7%)
ICAST-Trial	Meinck et al. (2018)	Very good (100.0%)	Very good (100.0%)	NR	NR	NR	Very good (91.7%)
IPPS	Gordon et al. (1979)	Adequate (55.6%)	Very good (77.8%)	Inadequate (25.0%)	Doubtful (26.7%)	NR	Adequate (54.1%)
MCNS	Lounds et al. (2004)	NR	Very good (100.0%)	NR	Adequate (73.3%)	NR	Very good (83.3%)
MCNS-SF	Lounds et al. (2004)	NR	Very good (77.8%)	NR	NR	Very good (100.0%)	Very good (83.3%)
P-CAAM	Rodriguez et al. (2011)	NR	Adequate (66.7%)	NR	NR	NR	Very good (89.2%)
POQ	Azar and Rohrbeck (1986)	NR	NR	NR	Doubtful (33.3%)	NR	Very good (77.8%)
	Haskett et al. (2006)	Doubtful (33.3%)	Very good (77.8%)	NR	NR	NR	Very good (82.8%)
	Mammen et al. (2003)	NR	NR	NR	NR	NR	Very good (77.3%)
PRCM	Vittrup et al. (2006)	NR	NR	NR	NR	NR	Very good (77.8%)
SBS-SV	Russell (2010)	NR	Very good (100.0%)	NR	NR	NR	NR

*Note.* AAPI-2 = Adult Adolescent Parenting Inventory–2; APT = Analog Parenting Task; CNQ = Child Neglect Questionnaire; CNS-MMS = Child Neglect Scales–Maternal Monitoring and Supervision scale; CTS-ES = Child Trauma Screen–Exposure Score; CTSPC = Conflict Tactics Scales: Parent–Child version; FM-CA = Family Maltreatment–Child Abuse criteria; ICAST-Trial = ISPCAN (International Society for the Prevention of Child Abuse and Neglect) Child Abuse Screening Tool for use in Trials; IPPS = Intensity of Parental Punishment Scale; MCNS = Mother–Child Neglect Scale; MCNS-SF = Mother–Child Neglect Scale–Short Form; P-CAAM = Parent–Child Aggression Acceptability Movie task; POQ = Parent Opinion Questionnaire; PRCM = Parental Response to Child Misbehavior questionnaire; SBS-SV = Shaken Baby Syndrome awareness assessment–Short Version.

^a^ Responsiveness was beyond the scope of this review; Measurement error is not displayed since it was not reported in any study; The methodological quality was rated using the consensus-based standards for the selection of health measurement instruments checklist (Mokkink, de Vet et al., 2018): very good, adequate, doubtful, and inadequate. The overall methodological quality per study was presented as a percentage of the ratings (Cordier et al., 2015): Inadequate = 0%–25%, Doubtful = 25.1%–50%, Adequate = 50.1%–75%, Very good = 75.1%–100%; NR = not reported (due to no psychometric data reported).

### Psychometric Properties and Quality of Evidence of the Instruments (Step 3)

Table 3 summarizes ratings for each psychometric property for single studies, respectively (Step 3.1). All data on a psychometric property extracted from the 25 included studies were evaluated against the criteria for good psychometric properties for the seven psychometric properties reported in this article (Prinsen et al., 2018). A summary of rating criteria is presented in detail in Supplementary Appendix B.

**Table 3.** Quality of the Psychometric Properties per Study.

		Psychometric Property: Quality of Psychometric Properties per Study^a^					
Instrument	Reference	Structural Validity	Internal Consistency	Cross-Cultural Validity	Reliability	Criterion Validity	Hypotheses Testing
AAPI-2	Bavolek and Keene (1999)	?	?	NR	NR	NR	±
	Conners et al. (2006)	−	?	NR	NR	NR	−
	Lawson et al. (2017)	±	?	NR	NR	NR	−
	Rodriguez et al. (2011)	NR	?	NR	NR	NR	±
	Russa and Rodriguez (2010)	NR	NR	NR	NR	NR	−
APT	Rodriguez et al. (2011)	NR	?	NR	NR	NR	−
	Russa and Rodriguez (2010)	NR	?	NR	NR	NR	±
CNQ	Stewart et al. (2015)	+	+	NR	NR	NR	−
CNS−MMS	Kirisci et al. (2001)	+	+	NR	NR	NR	−
CTS-ES	Lang and Connell (2017)	NR	NR	NR	NR	NR	±
CTSPC	Compier-de Block et al. (2017)	NR	?	NR	−	NR	+
	Cotter et al. (2018)	?	?	NR	NR	NR	−
	Grasso et al. (2016)	NR	?	NR	NR	NR	NR
	Kobulsky et al. (2017)	NR	NR	NR	?	NR	NR
	Lorber and Slep (2017)	?	?	NR	NR	NR	NR
	O’Dor et al. (2017)	NR	?	NR	NR	NR	−
	Rodriguez (2010)	NR	NR	NR	NR	NR	−
	Straus et al. (1998)	NR	?	NR	NR	NR	−
FM-CA	Heyman et al. (2019)	NR	NR	NR	NR	NR	?
ICAST-Trial	Meinck et al. (2018)	+	−	NR	NR	NR	−
IPPS	Gordon et al. (1979)	?	?	?	?	NR	±
MCNS	Lounds et al. (2004)	NR	?	NR	?	NR	−
MCNS-SF	Lounds et al. (2004)	NR	?	NR	NR	+	−
P-CAAM	Rodriguez et al. (2011)	NR	?	NR	NR	NR	±
POQ	Azar and Rohrbeck (1986)	NR	NR	NR	?	NR	+
	Haskett et al. (2006)	?	?	NR	NR	NR	−
	Mammen et al. (2003)	NR	NR	NR	NR	NR	−
PRCM	Vittrup et al. (2006)	NR	NR	NR	NR	NR	+
SBS-SV	Russell (2010)	NR	?	NR	NR	NR	NR

*Note*. AAPI-2 = Adult Adolescent Parenting Inventory–2; APT = Analog Parenting Task; CNQ = Child Neglect Questionnaire; CNS-MMS = Child Neglect Scales–Maternal Monitoring and Supervision Scale; CTS-ES = Child Trauma Screen–Exposure Score; CTSPC = Conflict Tactics Scales: Parent–Child version; FM-CA = Family Maltreatment–Child Abuse criteria; ICAST-Trial = ISPCAN (International Society for the Prevention of Child Abuse and Neglect) Child Abuse Screening Tool for use in Trials; IPPS = Intensity of Parental Punishment Scale; MCNS = Mother–Child Neglect Scale; MCNS-SF = Mother–Child Neglect Scale–Short Form; P-CAAM = Parent–Child Aggression Acceptability MOVIE TASK; POQ = Parent Opinion Questionnaire; PRCM = Parental Response to Child Misbehavior questionnaire; SBS-SV = Shaken Baby Syndrome Awareness Assessment–Short Version.

^a^ Responsiveness was beyond the scope of this review; Measurement error is not displayed since it was not reported in any study; The psychometric properties was rated using the criteria for good psychometric properties (Prinsen et al., 2018); + = sufficient; ? = indeterminate (due to less robust psychometric data); − = insufficient; ± = inconsistent (in case of rating one more results per psychometric property within a study, if < 75% of ratings displayed the same scoring); NR = not reported (due to no psychometric data); Data and ratings on each psychometric property per study are available in the Supplementary Appendix F.

Table 4 presents the overall ratings (Step 3.2) and the quality of evidence (Step 3.3) for each psychometric property per instrument; the results of all included studies on each psychometric property per instrument and their quality ratings are summarized in Supplementary Appendix F. None of the instruments reported overall ratings for all seven psychometric properties, given that measurement error was not reported (NR) for any of the 15 instruments. Furthermore, grades for quality of evidence were reported in only 21% (22 of 105 possible ratings) of all overall ratings on psychometric quality for all 15 instruments, while all other quality of evidence was rated as NR due to no psychometric data reported or not evaluated due to less robust psychometric data reported (i.e., indeterminate overall ratings).

**Table 4.** Overall Quality of Psychometric Properties and Evidence Quality per Instrument.

Instrument	Psychometric Property: Quality of Psychometric Properties and Quality of Evidence per Instrument											
	Structural Validity		Internal Consistency		Cross-Cultural Validity		Reliability		Criterion Validity		Hypotheses Testing	
	Overall Rating^a^	Quality of Evidence^b^	Overall rating^a^	Quality of Evidence^b^	Overall Rating^a^	Quality of Evidence^b^	Overall Rating^a^	Quality of Evidence^b^	Overall Rating^a^	Quality of Evidence^b^	Overall Rating^a^	Quality of Evidence^b^
AAPI-2	±	Moderate	?	NE	NR	NR	NR	NR	NR	NR	−	Moderate
APT	NR	NR	?	NE	NR	NR	NR	NR	NR	NR	±	Very Low
CNQ	+	Moderate	+	Low	NR	NR	NR	NR	NR	NR	−	High
CNS-MMS	+	High	+	High	NR	NR	NR	NR	NR	NR	−	Moderate
CTS-ES	NR	NR	NR	NR	NR	NR	NR	NR	NR	NR	±	Low
CTSPC	?	NE	?	NE	NR	NR	−	Moderate	NR	NR	−	High
FM-CA	NR	NR	NR	NR	NR	NR	NR	NR	NR	NR	?	NE
ICAST-Trial	+	High	−	High	NR	NR	NR	NR	NR	NR	−	High
IPPS	?	NE	?	NE	?	NE	?	NE	NR	NR	±	Low
MCNS	NR	NR	?	NE	NR	NR	?	NE	NR	NR	−	High
MCNS-SF	NR	NR	?	NE	NR	NR	NR	NR	+	High	−	High
P-CAAM	NR	NR	?	NE	NR	NR	NR	NR	NR	NR	±	Low
POQ	?	NE	?	NE	NR	NR	?	NE	NR	NR	−	High
PRCM	NR	NR	NR	NR	NR	NR	NR	NR	NR	NR	+	High
SBS-SV	NR	NR	?	NE	NR	NR	NR	NR	NR	NR	NR	NR

*Note.* AAPI-2 = Adult Adolescent Parenting Inventory–2; APT = Analog Parenting Task; CNQ = Child Neglect Questionnaire; CNS-MMS = Child Neglect Scales–Maternal Monitoring and Supervision scale; CTS-ES = Child Trauma Screen–Exposure Score; CTSPC = Conflict Tactics Scales: Parent–Child version; FM-CA = Family Maltreatment–Child Abuse criteria; ICAST-Trial = ISPCAN (International Society for the Prevention of Child Abuse and Neglect) Child Abuse Screening Tool for use in Trials; IPPS = Intensity of Parental Punishment Scale; MCNS = Mother–Child Neglect Scale; MCNS-SF = Mother–Child Neglect Scale–Short Form; P-CAAM = Parent–Child Aggression Acceptability Movie task; POQ = Parent Opinion Questionnaire; PRCM = Parental Response to Child Misbehavior questionnaire; SBS-SV = Shaken Baby Syndrome awareness assessment–Short Version.

^a^ The overall quality of psychometric properties was rated using the criteria for good psychometric properties (Mokkink, Prinsen, et al., 2018); + = sufficient rating; ? = indeterminate rating (due to less robust psychometric data); − = insufficient rating; ± = inconsistent rating; NR = not reported (due to no psychometric data); Data and ratings on each psychometric property per instrument are available in the Supplementary Appendix F. ^b^ The quality of evidence (confidence level for the overall quality rating of each psychometric property) was rated using a modified GRADE approach (Mokkink, Prinsen, et al., 2018): High = high level of confidence, Moderate = moderate level of confidence, Low = low level of confidence, Very Low = very low level of confidence, NR = not reported (due to not reported overall rating of psychometric properties); NE = not evaluated (due to indeterminate overall rating); If the evidence quality is very low, we should be concerned about using the overall ratings alone to recommend good instruments; Reasons for each grading on quality of evidence are available in the Supplementary Appendix F.

### Recommendations for the Most Suitable Instruments to Measure CM (Step 4)

Table 5 provides the recommendations for the use of parent or carer report instruments to measure CM in the future. None of instruments were rated as the most suitable; nine instruments (AAPI-2, APT, CNS-MMS, CTS-ES, FM-CA, IPPS, P-CAAM, PRCM, and SBS-SV) were considered the most promising but would still need further validation studies; six instruments (CNQ, CTSPC, ICAST-Trial, MCNS, MCNS-SF, and POQ), however, were not recommendable.

**Table 5.** Recommendations on Suitable Instruments for Their Future Use Adapted From Prinsen et al. (2018).

Category	Description on Category (Criteria)	Instruments	
A: Most suitable	Instruments that have the potential to be recommended for use in respect of the construct and population of interest (*instruments with high-quality evidence for sufficient content validity in any aspects of and at least low-quality evidence for sufficient internal consistency*)	None	
B: Promising but need further validation study	Instruments that may have the potential to be recommended for use, but further validation studies are needed (*instrument categorised not in A or C*)	AAPI-2 APT CNS-MMS CTS-ES FM-CA	IPPS P-CAAM PRCM SBS-SV
C: Not recommendable	Instruments that should not be recommended for use (*instruments with high-quality evidence for an insufficient psychometric property*)	CNQ CTSPC ICAST-Trial	MCNS MCNS-SF POQ

*Note.* AAPI-2 = Adult Adolescent Parenting Inventory–2; APT = Analog Parenting Task; CNQ = Child Neglect Questionnaire; CNS-MMS = Child Neglect Scales–Maternal Monitoring and Supervision scale; CTS-ES = Child Trauma Screen–Exposure Score; CTSPC = Conflict Tactics Scales: Parent–Child version; FM-CA = Family Maltreatment–Child Abuse criteria; ICAST-Trial = ISPCAN (International Society for the Prevention of Child Abuse and Neglect) Child Abuse Screening Tool for use in Trials; IPPS = Intensity of Parental Punishment Scale; MCNS = Mother–Child Neglect Scale; MCNS-SF = Mother–Child Neglect Scale–Short Form; P-CAAM = Parent–Child Aggression Acceptability Movie task; POQ = Parent Opinion Questionnaire; PRCM = Parental Response to Child Misbehavior questionnaire; SBS-SV = Shaken Baby Syndrome Awareness Assessment–Short Version.

## Discussion

The purpose of this systematic review was to evaluate the quality of psychometric properties (other than content validity and responsiveness) of all current parent/caregiver report instruments on CM by parents or caregivers and recommend the most suitable of these instruments using the COSMIN guidelines. This review identified 15 instruments and 25 studies on psychometric properties of these instruments. In general, the methodological quality of included studies was adequate. However, most of the identified instruments (12/15) reported on only three or less psychometric properties of the seven properties under review. Furthermore, there are limited high-quality evidence to suggest that any of the psychometric properties are inherently sufficient or insufficient. Therefore, most CM instruments (9/15) have the potential to be used in research and in clinical practice, but their psychometric quality should undergo further evaluation.

### Methodological Quality of the Included Studies

For structural validity, all but six instruments (AAPI-2, CNQ, CNS-MMS, CTSPC, ICAST-Trial, and IPPS) did not report any psychometric data or reported doubtful study quality. The doubtful study quality is due to using a less preferred factor analysis method, such as the exploratory factor analysis (EFA). The EFA can be used to identify a factor structure of new instruments without any prior hypothesis of the structure, while structural validity is to test a hypothesized factor structure of existing instruments (Mokkink, Prinsen, et al., 2018). To test the hypothesized factor structure, confirmative factor analysis (CFA) or item response theory (IRT) analysis was preferred in the COSMIN Risk of Bias checklist (Mokkink, de Vet et al., 2018). While having the same overall purpose for testing how well the data fit a predetermined factor structure (de Vet et al., 2011), the specific concerns of each analysis differ. That is, CFA focuses on total summed scores or responses because it assumes each item is equally weighted in terms of difficulty, whereas IRT analysis is concerned with individual responses to items under the assumption individual items may have different difficulty level (Lo et al., 2015). However, neither of these two analyses had been conducted for the factor structure of 10 instruments (APT, CTS-ES, FM-CA, IPPS, MCNS, MCNS-SF, P-CAAM, POQ, PRCM, and SBS-SV).

None of the instruments reported on all three psychometric properties within the domain of reliability (Mokkink et al., 2010b). Only four instruments (CTSPC, IPPS, MCNS, and POQ) reported reliability, while all but three instruments (CTS-ES, FM-CA, and PRCM) reported internal consistency. Even though measurement error is clinically very relevant information, none of the instruments reported measurement error. This is an important limitation to note as instruments with low error are able to detect clinically important changes sensitively and help clinicians to decide when to adjust treatment plans or to terminate treatment if the intervention has shown to have successfully addressed the underlying problem (Dvir, 2015; Guyatt et al., 1987). Consequently, the lack of reporting on all three of these psychometric properties makes it difficult to grasp overall reliability for all instruments comprehensibly.

Only one instrument (MCNS-SF) reported criterion validity between the shortened and an original (long) version; the MCNS-SF received a very good score for study quality. As there is no universally accepted gold standard to measure CM (Bailhache et al., 2013), this aspect of criterion validity could not be reported on in this review. In addition, cross-cultural validity for different demographic groups was reported for only one instrument (IPPS), with an inadequate score for study quality due to not reporting information on what kinds of factor analysis was used, despite comparing factor structures between mother and father respondents. Among culturally different groups using the same language, the same question may be interpreted differently. For example, “spanking” (as the most common form of corporal punishment) may be perceived as child abuse to parents in New Zealand but as discipline to American parents because corporal punishment is illegal (in all settings) in New Zealand but is legal if done at home in American (Elgar et al., 2018). This difference in interpretations between countries that speak the same language but show cultural differences may result in different underlying factor structures of the same instrument. For this reason, applying the same instruments to culturally different groups also requires testing measurement invariance across the different groups, even if they speak the same language.

Hypothesis testing for construct validity was reported for all instruments with ratings of either adequate or very good quality, except for the following two instruments: FM-CA received doubtful rating, and SBS-SV was NR. Seven instruments (APT, CNS-MMS, CTS-ES, FM-CA, ICAST-Trial, MSCNS, and MCNS-SF) reported on convergent validity only, calculating correlations between the scores of the seven instruments and a comparator CM instrument. One instrument (PRCM) reported on discriminative validity only, analyzing statistical differences in scores between parents who perpetrated CM and parents who did not. For six instruments (AAPI-2, CNQ, CTSPC, IPPS, P-CAAM, and POQ), both convergent and discriminative validity were reported. Except these six instruments, the imbalance between convergent and discriminative validity of the remaining instruments, therefore, has limited evidence for construct validity.

### Psychometric Properties of the Instruments

The evidence on structural validity is a prerequisite for interpreting the evidence on internal consistency (i.e., the interrelatedness of items in each scale or subscale; Mokkink, Prinsen, et al., 2018; Prinsen et al., 2018). For example, if results on structural validity show that a scale has four factors, internal consistency of each of those four subscales is more relevant than that of the total scale. As such, evidence on structural validity directly affected the overall ratings of internal consistency. Of the 12 instruments reporting evidence on internal consistency, only two instruments (CNQ and CNS-MMS) displayed sufficient internal consistency, CNQ with moderate evidence (due to only one adequate study available) for sufficient structural validity and high Cronbach’s α values and CNS-MMS with high evidence (due to very good study quality, consistent results, adequate sample sizes, and same populations between studies) for sufficient structural validity and a high Cronbach’s α. Conversely, five instruments (APT, MCNS, MCNS-SF, P-CAAM, and SBS-SV) did not report any data on structural validity; three instruments (CTSPC, IPPS, and POQ) reported indeterminate structural validity due to using a less robust factor analysis (EFA) or presenting only incomplete information on the structure of the instruments; one instrument (AAPI-2) reported conflicting results on the factor structure between studies. As these nine instruments (AAPI-2, APT, CTSPC, IPPS, MCNS, MCNS-SF, P-CAAM, POQ, and SBS-SV) demonstrated poor structural validity by not meeting the criteria of “at least low evidence for sufficient structural validity,” their internal consistency was therefore rated as indeterminate. Although one instrument (ICAST-Trial) reported high evidence for sufficient structural validity, internal consistency of the instrument was rated as insufficient due to a low Cronbach’s α.

Of four instruments reporting the evidence on reliability (test–retest, interrater, and intrarater reliability), three instruments (IPPS, MCNS, and POQ) gained indeterminate overall ratings because of reporting other reliability statistics (e.g., Spearman’s correlation coefficients and κ) than the preferred reliability statistics in the COSMIN criteria for good psychometric properties (Prinsen et al., 2018). The COSMIN criteria prefer the intraclass correlation coefficient (ICC) or the weighted κ as appropriate reliability statistics because in contrast to the Spearman’s ρ coefficient, the ICC takes into account systematic error caused by different conditions and learning effects in repeated measurements for continuous scales (Scholtes et al., 2011); the weighted κ takes into account the degree of disagreement between two raters for categorical scales whereas the unweighted κ does not (Tang et al., 2015). Although one instrument (CTSPC) reported ICC, reliability of the instrument was rated as insufficient (due to the ICC below the criterion for good reliability) with moderate evidence quality (due to some evidence from different population such as children).

Evidence on criterion validity of the shorten version of MCNS (MCNS-SF) was sufficient because the correlation with the original long version (MCNS) was over 0.70, which is the criterion for good criterion validity. In addition, evidence on cross-cultural validity was evaluated for only one instrument (IPPS), with an indeterminate overall rating, due to incomplete information on the measurement invariance of the instruments between two different groups. For good cross-cultural validity of an instrument, evidence on measurement invariance between culturally different groups (i.e., age, gender, language) should be found in factor structures at the scale level by performing CFA (Gregorich, 2006) or in item difficulty at item level by performing differential item functioning (DIF) analysis (Teresi et al., 2009). However, none of the instruments included in this review reported clear evidence on the measurement invariance between the different groups by using CFA or DIF analysis.

Evidence on hypothesis testing for construct validity was evaluated for all instruments except the SBS-SV. More than half of the instruments (8 of 15) reported insufficient hypothesis testing with high or moderate evidence quality: six instruments (CNQ, CTSPC, ICAST-Trial, MCNS, MCNS-SF, and POQ) had high-quality evidence while other two instruments (AAPI-2 and CNS-MMS) had moderate evidence (due to some evidence from different population such as university students who are not parents or caregivers). Conversely, only one instrument (PRCM) reported sufficient hypothesis testing with high-quality evidence. Four instruments (APT, CTS-ES, IPPS, and P-CAAM) reported conflicting results between studies on hypothesis testing, with low or very low evidence quality; only one instrument (FM-CA) reported indeterminate hypothesis testing due to using inappropriate statistical methods for comparison between FM-CA and a comparator CM instrument (i.e., calculating interrater agreement between two different measures rather than correlation). Furthermore, most hypothesis testing of instruments presented and considered only a *t*-value or *F*-value to confirm the statistical significance of the difference in scores between two groups (e.g., parents who perpetrated CM and parents who did not). However, these two statistics depend on sample size and do not account for the direction or magnitude of difference (Coe, 2002). To avoid this weakness of both statistics, this review converted the *t*-value or *F*-value to an effect size estimate (i.e., Cohen’s *d*) showing the direction and magnitude of differences between two groups regardless of sample sizes (Friedman, 1968; Thalheimer & Cook, 2002); an effect size of 0.5 or higher was used as a criterion for sufficient hypothesis testing on group differences. For this reason, some of the hypotheses, which were originally confirmed based on the *t*-value or *F*-value in the studies on hypothesis testing of the instruments, were rejected (insufficient rating) in our review based on the converted Cohen’s *d*.

### Recommendation of the Instruments (Step 4)

None of the included instruments have the potential to be recommended as the most suitable (category A) due to no high-quality evidence for sufficient content validity in a companion paper (Part 1; Yoon et al., 2020) and no at least low-quality evidence for sufficient internal consistency in this article (Part 2), while six instruments (CNQ, CTSPC, ICAST-Trial, MCNS, MCNS-SF, and POQ) should not be recommended at all (category C) due to high-quality evidence for insufficient hypotheses testing or internal consistency. As having no high-quality evidence for an insufficient psychometric property, nine instruments (AAPI-2, APT, CNS-MMS, CTS-ES, FM-CA, IPPS, P-CAAM, PRCM, and SBS-SV) may have potential to be recommended but need further validation studies (category B).

For each of the nine promising instruments, further validation studies on one or more properties are needed to determine whether the nine promising instruments could be recommendable (i.e., category A). As a criterion for category A, content validity, internal consistency, and/or structural validity (not the criterion but as a prerequisite for internal consistency) of all nine instruments should be further evaluated as a priority. In a companion paper (Part 1; Yoon et al., 2020), no high-quality evidence for content validity of any promising instruments (except FM-CA) was found due to missing data or lack of robust evidence in the content validity studies. For this reason, future studies on content validity may provide additional information and result in changed overall quality ratings of evidence for content validity. In addition, the internal consistency of most instruments (except CNS-MMS) was scored as NR due to no information of their internal consistency or indeterminate (?) due to no information of their structural validity. As such, the CTS-ES and PRCM require urgently further studies on their content validity, structural validity, and internal consistency due to no high-quality evidence on these psychometric properties; the AAPI-2, APT, CTS-ES, IPPS, P-CAAM, PRCM, and SBS-SV require further studies on their content validity and structural validity due to no high evidence for content validity and indeterminate internal consistency caused by unclarity around the unidimensionality of a scale or subscale (i.e., indeterminate or conflicting structural validity); the CNS-MMS requires further content validity studies due to no high evidence for content validity and high evidence for sufficient internal consistency; and the FM-CA requires further studies on its structural validity and internal consistency due to no evidence for these psychometric properties.

To confirm whether the six instruments (CNQ, CTSPC, ICAST-Trial, MCNS, MCNS-SF, and POQ) should indeed not be recommended, further validation studies on hypotheses testing and/or internal consistency need to be conducted. All six instruments were categorized into “not recommendable” (category C) due to high-quality evidence for insufficient hypotheses testing, while ICAST-Trial had high evidence for insufficient internal consistency—another reason for not being recommended. However, most hypotheses testing focused on comparisons between different instruments (convergent validity) rather than differences between groups (discriminative validity): that is, the ratio between the amount of hypotheses on convergent validity and discriminative validity is 5–1 in the CNQ; 7–5 in the CTSPC; 1–0 in the ICAST-Trial; 3–0 in the MCNS; 3–0 in the MCNS-SF; and 14–4 in the POQ. As the vast majority of evidence were based on convergent validity, hypotheses testing of the six instruments showed mostly one side of hypotheses testing without data on discriminative validity. To capture the overall picture of hypotheses testing, further discriminative validity studies of the six instruments are needed. These additional studies may change the assessment of the five of the six instruments (except ICAST-Trial) from not recommendable (category C) to promising (category B). In the case of ICAST-Trial, further studies on both hypotheses testing and internal consistency are needed.

### Limitations

This systematic review has some limitations. First of all, only instruments validated in English and studies published in English were included. Thus, some findings on psychometric properties of CM instruments published in other languages may have been excluded. Secondly, this review did not report on all of nine psychometric properties of the COSMIN taxonomy (Mokkink et al., 2010b); responsiveness was not considered for this review because evaluation of responsiveness would require to review all studies that have used the identified instruments as an outcome measure and would require a different search strategy altogether. Lastly, interpretability and feasibility were outside the scope of this article because they are not considered to be psychometric property according to the COSMIN taxonomy, even though these two instrument characteristics should be considered when recommending the most suitable instruments (Mokkink, Prinsen, et al., 2018; Prinsen et al., 2018). From a feasibility perspective, ideally instruments should have the least amount of items required to fully capture the construct under investigation to reduce the response time, particularly when it comes to investigating sensitive issues such as CM.

### Implication for Future Research

For researchers who want to comprehensively understand the overall psychometric properties of all current parent- or carer-reported CM instruments, this systematic review highlights the need for further validation studies of the instruments. Regarding structural validity, future factor analyses using CFA or IRT are needed for nine instruments (AAPI-2, APT, CTSPC, IPPS, MCNS, MCNS-SF, P-CAAM, POQ, and SBS-SV) to determine the quality of internal consistency of these nine instruments. To gain a comprehensive picture of reliability, all three elements of reliability should be assessed: internal consistency for CTS-ES, FM-CA, and PRCM; reliability (test–retest, interrater, and intrarater) for AAPI-2, APT, CNQ, CNS-MMS, CTS-ES, FM-CA, ICAST-Trial, MCNS-SF, P-CAAM, PRCM, and SBS-SV; and measurement error for all 15 instruments. In particular, ICC or weighted κ are required to be calculated and reported in future studies for test–retest, interrater, and intrarater reliability, rather than Spearman’s ρ or κ. With respect to cross-cultural validity, all 15 instruments (including IPPS with indeterminate cross-cultural validity) are needed to test measurement invariance across culturally different groups by performing CFA (Gregorich, 2006) or DIF analysis (Teresi et al., 2009). More hypothesis testing for construct validity should be conducted to determine convergent validity of the FM-CA, PRCM, and SBS-SV, and discriminative validity of the APT, CNS-MMS, CTS-ES, FM-CA, ICAST-Trial, MCNS, MCNS-SF, and SBS-SV. In particular, discriminative validity regarding differences in scores between groups should be based on the calculation of effect sizes such as Cohen’s *d* rather than *t*-values or *F*-values.

Apart from the suggestion of further validation studies on the psychometric properties of the identified instruments, the current results in this review support the need of future instrument development research of new parent/carer report instruments on CM as none of the included instruments on CM in this review could be identified or recommended as best instrument; and suggest some implications for the future development of a good instruments on CM. For good content validity as the most important psychometric property (Terwee et al., 2018), the items of a new instrument should be identified by an interview or survey with parents/carers to reflect respondents’ perspective on CM. This interview or survey with respondents was rarely done in the development studies for the existing 15 instruments on CM according to the findings of review in a companion paper (Part 1; Yoon et al., 2020), thus having a negative impact on the content validity. Next, for good internal consistency as the second most important property, robust factor analysis such as CFA or IRT should be conducted to identify a clear factor structure (good structural validity) as a prerequisite for internal consistency according to the Risk of Bias checklist (Mokkink, de Vet et al., 2018). Thirdly, for good psychometric properties in general, appropriate statistics for each psychometric property need to be calculated and reported on, in accordance with the criteria for good psychometric properties (Prinsen et al., 2018). Lastly, for high-quality evidence on each psychometric property, new parent/carer report instruments on CM should be developed against the standards set out in the COSMIN Risk of Bias checklist (Mokkink, de Vet et al., 2018): that is, appropriate study design and robust statistical analysis would ensure good methodological quality (no concern regarding risk of bias), consistent results across the psychometric studies (no concern regarding inconsistency), precision of the evidence by using appropriate sample size (no concern regarding imprecision), and direct evidence from targeted population such as parents or caregivers (no concern regarding indirectness) in terms of evidence quality according to the GRADE approach (Prinsen et al., 2018).

## Conclusion

This systematic review evaluated the psychometric properties of 15 parent- or caregiver-reported CM instruments using the COSMIN guidelines. Evidence concerning psychometric properties was limited and mostly of lower quality. Based on current available psychometric evidence, none of the included instruments met the requirements to be recommended as most suitable instrument. Only nine instruments (AAPI-2, APT, CNS-MMS, CTS-ES, FM-CA, IPPS, P-CAAM, PRCM, and SBS-SV) were recommended as promising but would still need further validation before any possible recommendations as most suitable instrument may be made.

## Supplemental Material

Supplemental_Material - A Systematic Review Evaluating Psychometric Properties of Parent or Caregiver Report Instruments on Child Maltreatment: Part 2: Internal Consistency, Reliability, Measurement Error, Structural Validity, Hypothesis Testing, Cross-Cultural Validity, and Criterion Validity Click here for additional data file. Supplemental_Material for A Systematic Review Evaluating Psychometric Properties of Parent or Caregiver Report Instruments on Child Maltreatment: Part 2: Internal Consistency, Reliability, Measurement Error, Structural Validity, Hypothesis Testing, Cross-Cultural Validity, and Criterion Validity by Sangwon Yoon, Renée Speyer, Reinie Cordier, Pirjo Aunio and Airi Hakkarainen in Trauma, Violence, & Abuse
