# Supplementary material for: A Systematic Review Evaluating Psychometric Properties of Parent or Caregiver Report Instruments on Child Maltreatment: Part 2: Internal Consistency, Reliability, Measurement Error, Structural Validity, Hypothesis Testing, Cross-Cultural Validity, and Criterion Validity
Source: Trauma Violence Abuse. 2020 Apr 9;22(5):1296–315. doi: 10.1177/1524838020915591 (PMC8739544; doi:10.1177/1524838020915591)
Supplement: Supplemental_Material - A Systematic Review Evaluating Psychometric Properties of Parent or Caregiver Report Instruments on Child Maltreatment: Part 2: Internal Consistency, Reliability, Measurement Error, Structural Validity, Hypothesis Testing, Cross-Cultural Validity, and Criterion Validity [file Supplemental_Material.zip › Appendix A.pdf]

Supplementary Appendices

Appendix A. *Database Search Strategies.*

Database	Search Terms (Subject heading and Free text words)	Number of records
**CINAHL**	(((MH "Child Abuse+") OR (MH "Domestic Violence+") OR (MH "Family Conflict") OR (MH "Aggression+") OR (MH "Punishment")) AND ((MH "Parents+") OR (MH "Parenting") OR (MH "Father-Infant Relations")OR (MH "Father-Child Relations") OR (MH "Fathers+") OR (MH "Mother-Child Relations") OR (MH "Mother-Infant Relations") OR (MH "Mothers+") OR (MH "Family+") OR (MH "Caregivers") OR (MH "Child Rearing+")) AND ((MH "Psychometrics") OR (MH "Measurement Issues and Assessments") OR (MH "Validity") OR (MH "Predictive Validity") OR (MH "Reliability and Validity") OR (MH "Internal Validity") OR (MH "Face Validity") OR (MH "External Validity") OR (MH "Discriminant Validity") OR (MH "Criterion-Related Validity") OR (MH "Consensual Validity") OR (MH "Concurrent Validity") OR (MH "Qualitative Validity") OR (MH "Construct Validity") OR (MH "Content Validity") OR (MH "Questionnaire Validation") OR (MH "Validation Studies") OR (MH "Test-Retest Reliability") OR (MH "Sensitivity and Specificity") OR (MH "Reproducibility of Results") OR (MH "Reliability") OR (MH "Intrarater Reliability") OR (MH "Interrater Reliability") OR (MH "Measurement Error") OR (MH "Bias (Research)") OR (MH "Selection Bias") OR (MH "Sampling Bias") OR (MH "Precision") OR (MH "Sample Size Determination") OR (MH "Repeated Measures") OR (Psychometric* or reliability or validit* or reproducibility or bias))) OR (((child OR children OR infant* OR toddler* OR neonate* OR baby OR babies OR adolescent* OR teen* OR minor*) AND (victim* OR aggress* OR punish* OR abus* OR maltreat* OR neglect* OR mistreat* or violen* or conflict* or batter* or molest*) AND (rear* OR parent* OR father* OR mother* OR family OR families OR domestic* OR caregiver* OR carer* OR caring OR home OR homes) AND (psychometric* OR reliabilit* OR validit* OR reproducibilit* OR bias)) Limiters - Published Date:20181001-20191031)	1,173
**Embase**	((child abuse/ OR child neglect/ OR emotional abuse/ OR physical abuse/ OR battering/ OR domestic violence/ OR physical violence/ OR family conflict/ OR victim/ OR aggression/ OR punishment/) AND (parent/ OR father/ OR father child relation/ OR mother/ OR mother child relation/ OR family/ OR caregiver/ OR child rearing/) AND (psychometry/ or validity/ or reliability/ or measurement error/ or measurement precision/ or measurement repeatability/ or error/ or statistical bias/ or test retest reliability/ or intrarater reliability/ or interrater reliability/ or accuracy/ or criterion validity/ or internal validity/ or face validity/ or external validity/ or discriminant validity/ or concurrent validity/ or qualitative validity/ or construct validity/ or content validity/)) OR (((child OR children OR infant* OR toddler* OR neonate* OR baby OR babies OR adolescent* OR teen* OR minor*) AND (victim* OR aggress* OR punish* OR abus* OR maltreat* OR neglect* OR mistreat* or violen* or conflict* or batter* or molest*) AND (rear* OR parent* OR father* OR mother* OR family OR families OR domestic* OR caregiver* OR carer* OR caring OR home OR homes) AND (psychometric* OR reliabilit* OR validit* OR reproducibilit* OR bias)) limit to yr="2019 -Current")	456
**ERIC**	((Child abuse/ OR Child neglect/ OR violence/ OR family violence/) AND (parenting styles/ OR parents/ OR child rearing/ OR father attitudes/ OR fathers/ OR mother attitudes/ OR mothers/ OR family attitudes/ OR caregiver attitudes/ OR caregiver child relationship/ OR caregiver role/ OR family environment/) AND (Psychometrics/ OR Validity/ OR Reliability/ OR Error of Measurement/ OR Bias/ OR Interrater Reliability/ OR Accuracy/ OR Predictive Validity/ OR Construct Validity/ OR Content Validity/)) OR (((child OR children OR infant* OR toddler* OR neonate* OR baby OR babies OR adolescent* OR teen* OR minor*) AND (victim* OR aggress* OR punish* OR abus* OR maltreat* OR neglect* OR mistreat* or violen* or conflict* or batter* or molest*) AND (rear* OR parent* OR father* OR mother* OR family OR families OR domestic* OR caregiver* OR carer* OR caring OR home OR homes) AND (psychometric* OR reliabilit* OR validit* OR reproducibilit* OR bias)) limit to yr="Last year")	523

(continued)

Appendix A. *(continued)*

Database	Search Terms (Subject heading and Free text words)	Number of records
**PsycINFO**	((child abuse/ OR child neglect/ OR violence/ OR domestic violence/ OR physical abuse/ OR family conflict/ OR victimization/ OR aggressive behaviOR/ OR aggressiveness/ OR punishment/) AND (parent child communication/ OR parent child relations/ OR parenting/ OR parenting style/ OR parents/ OR father child communication/ OR father child relations/ OR fathers/ OR mother child communication/ OR mother child relations/ OR mothers/ OR family/ OR caregivers/) AND (Psychometrics/ OR Statistical Validity/ OR Test Validity/ OR Statistical Reliability/ OR Test Reliability/ OR Error of Measurement/ OR Errors/ OR Response Bias/ OR Interrater Reliability/ OR Repeated Measures/)) OR (((child OR children OR infant* OR toddler* OR neonate* OR baby OR babies OR adolescent* OR teen* OR minor*) AND (victim* OR aggress* OR punish* OR abus* OR maltreat* OR neglect* OR mistreat* or violen* or conflict* or batter* or molest*) AND (rear* OR parent* OR father* OR mother* OR family OR families OR domestic* OR caregiver* OR carer* OR caring OR home OR homes) AND (psychometric* OR reliabilit* OR validit* OR reproducibilit* OR bias)) limit to yr="2019 -Current")	285
**PubMed**	(("Child Abuse"[Mesh] OR "Physical Abuse"[Mesh] OR "Domestic Violence"[Mesh] OR "Violence"[Mesh] OR "Family Conflict"[Mesh] OR "Aggression"[Mesh] OR "Punishment"[Mesh]) AND ("Parents"[Mesh] OR "Parent-Child Relations"[Mesh] OR "Parenting"[Mesh] OR "Fathers"[Mesh] OR "Father-Child Relations"[Mesh] OR "Mothers"[Mesh] OR "Mother-Child Relations"[Mesh] OR "Family"[Mesh] OR "Caregivers"[Mesh] OR "Child Rearing"[Mesh]) AND ("Psychometrics"[Mesh] OR "Reproducibility of Results"[Mesh] OR "Validation Studies as Topic"[Mesh] OR "Validation Studies" [Publication Type] OR "Bias"[Mesh] OR "Observer Variation"[Mesh] OR "Selection Bias"[Mesh] OR "Diagnostic Errors"[Mesh] OR "Dimensional Measurement Accuracy"[Mesh] OR "Predictive Value of Tests"[Mesh] OR "Discriminant Analysis"[Mesh])) OR (((child OR children OR infant* OR toddler* OR neonate* OR baby OR babies OR adolescent* OR teen* OR minor*) AND (victim* OR aggress* OR punish* OR abus* OR maltreat* OR neglect* OR mistreat* or violen* or conflict* or batter* or molest*) AND (rear* OR parent* OR father* OR mother* OR family OR families OR domestic* OR caregiver* OR carer* OR caring OR home OR homes) AND (psychometric* OR reliabilit* OR validit* OR reproducibilit* OR bias)) Filters: Publication date from 2018/10/05 to 2019/10/05)	1,092
**Sociological Abstracts**	(MAINSUBJECT.EXACT("Child Neglect") OR MAINSUBJECT.EXACT("Child Abuse") OR (MAINSUBJECT.EXACT("Violence") OR MAINSUBJECT.EXACT("Family Violence")) OR MAINSUBJECT.EXACT("Family Conflict") OR MAINSUBJECT.EXACT("Victimization") OR MAINSUBJECT.EXACT("Victims") OR MAINSUBJECT.EXACT("Aggression") OR (MAINSUBJECT.EXACT("Punishment") OR MAINSUBJECT.EXACT("Corporal Punishment")) OR MAINSUBJECT.EXACT("Emotional Abuse")) AND (MAINSUBJECT.EXACT("Parent Child Relations") OR MAINSUBJECT.EXACT("Parental Influence") OR MAINSUBJECT.EXACT("Parents") OR MAINSUBJECT.EXACT("Parental Attitudes") OR MAINSUBJECT.EXACT("Parenthood")) OR MAINSUBJECT.EXACT("Childrearing Practices") OR MAINSUBJECT.EXACT("Fathers") OR MAINSUBJECT.EXACT("Mothers") OR (MAINSUBJECT.EXACT("Family") OR MAINSUBJECT.EXACT("Family Relations") OR MAINSUBJECT.EXACT("Family Conflict") OR MAINSUBJECT.EXACT("Family Violence")) OR MAINSUBJECT.EXACT("Caregivers")) AND (MAINSUBJECT.EXACT("Psychometric Analysis") OR MAINSUBJECT.EXACT("Validity") OR MAINSUBJECT.EXACT("Reliability") OR MAINSUBJECT.EXACT("Error of Measurement") OR MAINSUBJECT.EXACT("Errors") OR MAINSUBJECT.EXACT("Test Bias") OR MAINSUBJECT.EXACT("Statistical Bias") OR MAINSUBJECT.EXACT("Bias") OR MAINSUBJECT.EXACT("Accuracy") OR MAINSUBJECT.EXACT("Agreement") OR MAINSUBJECT.EXACT("Research Design Error") OR MAINSUBJECT.EXACT("Specificity") OR MAINSUBJECT.EXACT("Sampling"))	133

Notes. All searches performed on the 29th of January 2018 with an update on the 5th of October 2019.
