# Supplementary material for: A Systematic Review Evaluating Psychometric Properties of Parent or Caregiver Report Instruments on Child Maltreatment: Part 2: Internal Consistency, Reliability, Measurement Error, Structural Validity, Hypothesis Testing, Cross-Cultural Validity, and Criterion Validity
Source: Trauma Violence Abuse. 2020 Apr 9;22(5):1296–315. doi: 10.1177/1524838020915591 (PMC8739544; doi:10.1177/1524838020915591)
Supplement: Supplemental_Material - A Systematic Review Evaluating Psychometric Properties of Parent or Caregiver Report Instruments on Child Maltreatment: Part 2: Internal Consistency, Reliability, Measurement Error, Structural Validity, Hypothesis Testing, Cross-Cultural Validity, and Criterion Validity [file Supplemental_Material.zip › Appendix B.pdf]

Appendix B. *Criteria for Good Psychometric Properties Adapted from Prinsen et al. (2018).*

Psychometric property	Rating[a]	Quality criteria
Structural validity	+	**CTT:** CFA: CFI or TLI or comparable measure > 0.95 OR RMSEA < 0.06 OR SRMR < 0.08 (e.g., If at least one of CFI and TLI > 0.95)<br>**IRT/Rasch:** CFI or TLI or comparable measure > 0.95 OR RMSEA < 0.06 OR SRMR < 0.08 AND residual correlations between the items after controlling for the dominant factor < 0.20 OR Q3's < 0.37 AND adequate looking graphs for monotonicity OR item scalability > 0.30 AND IRT x^2 > 0.01; Rasch: 0.5 ≤ infit and outfit mean squares ≤ 1.5 OR -2 < Z-standardised values < 2
	?	Not all information for '+' reported (e.g., **CTT:** If no psychometric data on any of CFI, TLI, RMSEA, or SRMR)
	-	Criteria for '+' not met (e.g., **CTT**: If both CFI and TLI ≤ 0.95)
	NR	No information found on structural validity
Hypotheses testing for construct validity	+	Correlations with instruments measuring similar constructs ≥ 0.50 OR meaningful differences between relevant (sub)groups (e.g., Cohen's d ≥ 0.50) OR at least 75% of the results are in accordance with the hypotheses
	?	Not all information for '+' reported (e.g., If only p-value and lack of information to calculate Cohen's d)
	-	Criteria for '+' not met (e.g., If Correlation r or Cohen's d < 0.50 or less than 75% of the results not in accordance with the hypotheses)
	NR	No information found on hypotheses testing for construct validity
Cross-cultural validity	+	No important differences found between group factors such as age, gender, and language in multiple group factor analysis OR DIF analysis: McFadden's R-Squared < 0.02
	?	Not all information for '+' reported (e.g., If no psychometric data on multiple group factor or DIF analysis)
	-	Criteria for '+' not met (e.g., If McFadden's R-Squared ≥ 0.02)
	NR	No information found on Cross-cultural validity\measurement invariance
Criterion validity	+	Correlation with gold standard ≥ 0.70 OR AUC ≥ 0.70
	?	Not all information for '+' reported (e.g., If no psychometric data on AUC)
	-	Criteria for '+' not met (e.g., if AUC < 0.70)
	NR	No information found on criterion validity
Measurement error	+	SDC or LoA < MIC
	?	Not all information for '+' reported (e.g., If no psychometric data on MIC)
	-	Criteria for '+' not met (e.g., If LoA ≥ MIC)
	NR	No information found on measurement error
Internal consistency	+	At least low evidence[b] for sufficient structural validity AND Cronbach's alpha(s) ≥ 0.70
	?	Not all information for '+' reported OR Criteria for "At least low evidence[b] for sufficient structural validity not met (e.g., If no psychometric data on Cronbach's alpha or very low evidence for sufficient structural validity regardless of Cronbach alpha)
	-	Criteria for '+' not met (e.g., If low evidence for sufficient structural validity but Cronbach's alpha < 0.70)
	NR	No information found on internal consistency
Reliability	+	ICC or weighted Kappa ≥ 0.70
	?	Not all information for '+' reported (e.g., If no psychometric data on ICC)
	-	Criteria for '+' not met (e.g., If ICC < 0.70)
	NR	No information found on reliability

Note. AUC = Area Under the Curve; CFA = Confirmatory Factor Analysis; CFI = Comparative Fit Index; CTT = Classical Test Theory; DIF = Differential Item Functioning; ICC = Intraclass Correlation Coefficient; IRT = Item Response Theory; LoA = Limits of Agreement; MIC = Minimal Important Change; RMSEA: Root Mean Square Error of Approximation; SEM = Standard Error of Measurement; SDC = Smallest Detectable Change; SRMR: Standardised Root Mean Residuals; TLI = Tucker-Lewis Index.

[a] + = Sufficient; - = Insufficient; ? = Indeterminate; ± = Inconsistent; NR = Not Reported.

[b] As defined by grading the evidence according to the GRADE approach (Mokkink, Prinsen, et al., 2018).
