# Supplementary material for: A Systematic Review Evaluating Psychometric Properties of Parent or Caregiver Report Instruments on Child Maltreatment: Part 2: Internal Consistency, Reliability, Measurement Error, Structural Validity, Hypothesis Testing, Cross-Cultural Validity, and Criterion Validity
Source: Trauma Violence Abuse. 2020 Apr 9;22(5):1296–315. doi: 10.1177/1524838020915591 (PMC8739544; doi:10.1177/1524838020915591)
Supplement: Supplemental_Material - A Systematic Review Evaluating Psychometric Properties of Parent or Caregiver Report Instruments on Child Maltreatment: Part 2: Internal Consistency, Reliability, Measurement Error, Structural Validity, Hypothesis Testing, Cross-Cultural Validity, and Criterion Validity [file Supplemental_Material.zip › Appendix C.pdf]

Appendix C. *Modified GRADE Approach for Rating the Quality of Evidence on Measurement Properties Adapted from Prinsen et al. (2018).*

**Level of evidence quality (sum of scores per factor)**	**Factor**	**Score**	**Criteria**
**High (0)**	*Risk of bias*	0	Multiple studies of at least adequate methodological quality<br>*OR*<br>One study of very good methodological quality
**Moderate (-1)**		-1	Multiple studies of doubtful methodological quality<br>*OR*<br>Only one study of adequate methodological quality
**Low (-2)**		-2	Multiple studies of inadequate methodological quality<br>*OR*<br>Only one study of doubtful methodological quality
**Very low (< -3)**		-3	Only one study of inadequate methodological quality
	Inconsistency	0	All studies show the same results
		-1	Less than 75% of studies show either sufficient or insufficient results
		-2	50% of studies displayed sufficient results against the criteria<br>*AND*<br>Other 50% of studies displayed insufficient results against the criteria
	Imprecision	0	Total sample size > 100
		-1	Total sample size = 50–100
		-2	Total sample size = n < 50
	Indirectness	0	All studies addressing construct or target population of the review
		-1	At least one study not addressing construct or target population of the review, but not all
		-2	All studies not addressing construct or target population of the review

Note. The starting point of evidence quality is 'high' quality of evidence; the level of evidence quality is downgraded by the sum of scores per factors.
