# Supplementary material for: A Systematic Review Evaluating Psychometric Properties of Parent or Caregiver Report Instruments on Child Maltreatment: Part 2: Internal Consistency, Reliability, Measurement Error, Structural Validity, Hypothesis Testing, Cross-Cultural Validity, and Criterion Validity
Source: Trauma Violence Abuse. 2020 Apr 9;22(5):1296–315. doi: 10.1177/1524838020915591 (PMC8739544; doi:10.1177/1524838020915591)
Supplement: Supplemental_Material - A Systematic Review Evaluating Psychometric Properties of Parent or Caregiver Report Instruments on Child Maltreatment: Part 2: Internal Consistency, Reliability, Measurement Error, Structural Validity, Hypothesis Testing, Cross-Cultural Validity, and Criterion Validity [file Supplemental_Material.zip › Appendix D.pdf]

Appendix D. *Overview of Child Maltreatment Instrument: Reasons for Exclusion.*

No	Instrument (References)[a]	Abbreviation	Reason for exclusion
1	Adolescent Clinical Sexual Behavior Inventory (William N. Friedrich, Lysne, Sim, & Shamos, 2004)	ACSBI	Not a measure of child maltreatment
2	Adolescent Sexual Behavior Inventory–Self report (Wherry, Berres, Sim, & Friedrich, 2009)	ACSBI-S	Not a measure of child maltreatment
3	Adult Attachment Interviews (Hesse, 2008)	AAIs	Not a parent-report measure
4	Adult–Adolescent Parenting Inventory (Bavolek, 1984)	AAPI	Old version of a revised measure
5	Adverse Childhood Experiences questionnaire (Felitti et al., 1998)	ACEs	Not a parent-report measure
6	Alabama Parenting Questionnaire (Shelton, Frick, & Wootton, 1996)	APQ	Not a measure of child maltreatment
7	Assessing Environments III (Berger, Knutson, Mehm, & Perkins, 1988)	AEIII	Not a parent-report measure
8	Assessment of parental awareness of the shaken baby syndrome[b] (Mann, Rai, Sharif, & Vavasseur, 2015)	N/A	No psychometric data found
9	Body Image Victimization Experiences Scale (Duarte & Pinto-Gouveia, 2017)	BIVES	Not a measure of child maltreatment
10	Brief Child Abuse Potential Inventory (Ondersma, Chaffin, Mullins, & LeBreton, 2005)	BCAP	Not a measure of child maltreatment
11	Brigid Collins Risk Screener (Weberling, Forgays, Crain-Thoreson, & Hyman, 2003)	BCRS	Not a measure of child maltreatment
12	California Family Risk Assessment (W. L. Johnson, 2011)	CFRA	Not a parent-report measure
13	Caregiver–Child Social/Emotional and Relationship Rating Scale (McCall, Groark, & Fish, 2010)	CCSERRS	Not a measure of child maltreatment
14	CHild Abuse InveNtory at Emergency Rooms (Sittig et al., 2016)	CHAINER	Not a parent-report measure
15	Child Abuse Potential Inventory (Milner, 1986)	CAP	Not a measure of child maltreatment
16	Child Abuse Risk Assessment Scale (Chan, 2012)	CARAS	Not developed in English
17	Child and Adolescent Trauma Screen (Sachser et al., 2017)	CATS	Not a measure of child maltreatment
18	Child Behavior CheckList (Achenbach & Rescorla, 2000)	CBCL	Not a measure of child maltreatment
19	Child emotional maltreatment module[b] (A. M. Slep, Heyman, & Snarr, 2011)	N/A	No psychometric data found
20	Child maltreatment assessment (Salum et al., 2016)	N/A	Not developed in English
21	Child maltreatment measure[b] (Tajima, Herrenkohl, Huang, & Whitney, 2004)	N/A	No psychometric data found
22	Child Protective Services Review Document (Fanshel, Finch, & Grundy, 1994)	CPSRD	Not a parent-report measure
23	Child Reflective Functioning scale (Ensink et al., 2015)	CRF	Not a measure of child maltreatment
24	Child Sexual Behavior Inventory (W. N. Friedrich et al., 2001)	CSBI	Not a measure of child maltreatment
25	Child Well-Being Scales (Gaudin, Polansky, & Kilpatrick, 1992)	CWBS	Not a parent-report measure
26	Childhood Experience of Care and Abuse (Brown, Craig, Harris, Handley, & Harvey, 2007)	CECA	Not a parent-report measure
27	Childhood Experience of Care and Abuse Questionnaire (N. Smith, Lam, Bifulco, & Checkley, 2002)	CECA.Q	Not a parent-report measure
28	Childhood Experiences of Violence Questionnaire (Walsh, MacMillan, Trocme, Jamieson, & Boyle, 2008)	CEVQ	Not a parent-report measure
29	Childhood Trauma Interview (Fink, Bernstein, Handelsman, Foote, & Lovejoy, 1995)	CTI	Not a parent-report measure
30	Childhood Trauma Questionnaire (Bernstein, Ahluvalia, Pogge, & Handelsman, 1997)	CTQ	Not a parent-report measure
31	Childhood Trauma Questionnaire Short Form (Forde, Baron, Scher, & Stein, 2012)	CTQ-SF	Not a parent-report measure
32	Child–Parent Relationship Scale (Driscoll & Pianta, 2011)	CPRS	Not a measure of child maltreatment
33	Child–Parent Relationship Scale–Short Form (Pianta, 1992)	CPRS-SF	Not a measure of child maltreatment
34	Children Intimate Relationships, and Conflictual Life Events interview (Marshall, Feinberg, Jones, & Chote, 2017)	CIRCLE	Not a parent-report measure
35	Children's Impact of Traumatic Events Scale–Revised (Chaffin & Shultz, 2001)	CITES-R	Not a measure of child maltreatment
36	Christchurch trauma assessment (Nelson, Lynskey, Heath, & Martin, 2010)	N/A	Not a parent-report measure
37	Cleveland Child Abuse Potential Scale (Ezzo & Young, 2012)	C-CAPS	Not a parent-report measure
38	Comprehensive Childhood Maltreatment Inventory (Riddle & Aponte, 1999)	CCMI	Not a parent-report measure
39	Conflict Tactic Scale 2 (Straus et al., 2003)	CTS 2	Not a measure of child maltreatment

(Continued)

Appendix D. *(continued)*

No	Instrument (References)[a]	Abbreviation	Reason for exclusion
40	Conflict Tactics Scales (Straus et al., 2003)	CTS	Not a measure of child maltreatment
41	Defense Style Questionnaire (Bond & Wesley, 1996)	DSQ	Not a parent-report measure
42	Disciplinary methods interview[b] (Thompson, 2017)	N/A	Not a measure of child maltreatment
43	Discipline survey (Socolar, Savage, Devellis, & Evans, 2004)	N/A	Not a measure of child maltreatment
44	Dunedin Family Services Indicator (Muir et al., 1989)	DFSI	Not a parent-report measure
45	Dyadic Parent–child Interaction Coding System-II (Eyberg, Bessmer, Newcomb, Edwards, & Robinson, 1994)	DPICS-II	Not a parent-report measure
46	Egna Minnen Beträffande Uppfostran (My Memories of Upbringing) (Castro, de Pablo, Gomez, Arrindell, & Toro, 1997)	EMBU	Not developed in English
47	Egna Minnen Betrffånde Uppfostran for Children (Castro et al., 1997; Markus, Lindhout, Boer, Hoogendijk, & Arrindell, 2003)	EMBU-C	Not a parent-report measure
48	Emotional and Physical Abuse Questionnaire (Kemper, Carlin, & Buntain-Ricklefs, 1994)	EPAB	Not a parent-report measure
49	Environmental harshness, health, and life history strategy Indicators[b] (Chua, Lukaszewski, Grant, & Sng, 2017)	N/A	Not a measure of child maltreatment
50	Exposure To community Violence (Richters & Martinez, 1993)	ETV	Not a measure of child maltreatment
51	Exposure to violence questionnaire[b] (Kuo, Mohler, Raudenbush, & Earls, 2000)	N/A	Not a measure of child maltreatment
52	Familial Experiences Questionnaire (Wheelock, Lohr, & Silk, 1997)	FEQ	Not a parent-report measure
53	Family Affective Attitude Rating Scale (Waller, Gardner, Dishion, Shaw, & Wilson, 2012)	FAARS	Not a measure of child maltreatment
54	Family Aggression Screening Tool (Cecil, McCrory, Viding, Holden, & Barker, 2016)	FAST	Not a parent-report measure
55	Family Background Questionnaire–Brief (Melchert & Kalemeera, 2009)	FBQ-B	Not a parent-report measure
56	Family Behaviors Screen (Simmons, Craun, Farrar, & Ray, 2017)	FBS	Not a measure of child maltreatment
57	Family Betrayal Questionnaire (Delker, Smith, Rosenthal, Bernstein, & Freyd, 2017)	FBQ	Not a measure of child maltreatment
58	Family Law Detection Of Overall Risk Screen (McIntosh, Wells, & Lee, 2016)	FL-DOORS	Not a measure of child maltreatment
59	Family maltreatment diagnostic criteria (Heyman & Smith Slep, 2009)	N/A	Not a parent-report measure
60	Family Risk of Abuse And Neglect (Lennings, Brummert Lennings, Bussey, & Taylor, 2014)	FRAAN	Not a measure of child maltreatment
61	Family Therapy Alliance Scale (L. N. Johnson, Ketring, & Anderson, 2013)	FTAS	Not a measure of child maltreatment
62	Family Unpredictability Scale (Ross & Hill, 2000)	FUS	Not a measure of child maltreatment
63	Go/No–go Association Task Physical Discipline (Sturge-Apple, Rogge, Peltz, Suor, & Skibo, 2015)	GNAT-Physical Discipline	Not a measure of child maltreatment
64	Home Observation Measure of the Environment (Caldwell & Bradley, 2003)	HOME	Not a parent-report measure
65	Home safety screening (Scribano, Stevens, Marshall, Gleason, & Kelleher, 2011)	N/A	Not a measure of child maltreatment
66	Identification of Parents At Risk for child Abuse and Neglect (van der Put et al., 2017)	IPARAN	Not developed in English
67	Index of Child Care Environment (Anme et al., 2013)	ICCE	Not developed in English
68	Invalidating Childhood Environments Scale (Mountford, Corstorphine, Tomlinson, & Waller, 2007)	ICES	Not a measure of child maltreatment
69	Inventory on beliefs and attitudes towards domestic violence (Hutchinson & Doran, 2017)	N/A	Not a measure of child maltreatment
70	ISPCAN Child Abuse Screening Tool Children's version (Zolotor et al., 2009)	ICAST-C	Not a parent-report measure
71	ISPCAN Child Abuse Screening Tool Parents' version (Runyan et al., 2009)	ICAST-P	Developed in multiple languages
72	ISPCAN Child Abuse Screening Tools Retrospective version (Dunne et al., 2009)	ICAST-R	Not a parent-report measure
73	Japanese version of Conflict Tactics Scale[b] (Baba et al., 2017)	CTS1: Japanese version	Developed in English but translated and validated in other languages
74	Juvenile Victimization Questionnaire (Finkelhor, Hamby, Ormrod, & Turner, 2005)	JVQ	Not a parent-report measure

(Continued)

Appendix D. *(continued)*

No	Instrument (References)[a]	Abbreviation	Reason for exclusion
75	Maternal Characteristics Scale (Polansky, Gaudin, & Kilpatrick, 1992)	MCS	Not a measure of child maltreatment
76	Maternal discipline and appropriateness[b] (Padilla-Walker, 2008)	N/A	Not a parent-report measure
77	Maternal Responsiveness Questionnaire (Leerkes & Qu, 2017)	MRQ	Not a measure of child maltreatment
78	Maternal Self-report Support Questionnaire (D. W. Smith et al., 2010)	MSSQ	Not a measure of child maltreatment
79	Maternal Support Questionnaire–Child Report (D. W. Smith et al., 2017)	MSQ-CR	Not a measure of child maltreatment
80	Meaning of the Child interview (Grey & Farnfield, 2017)	MotC	Not a measure of child maltreatment
81	Measure Of Parenting Style (Parker et al., 1997)	MOPS	Not a parent-report measure
82	MeaSure trauma associated with Child Sexual Abuse (Choudhary, Satapathy, & Sagar, 2018)	MSCSA	Not a measure of child maltreatment
83	Measures of community–relevant outcomes for violence prevention programs[b] (Hausman et al., 2013)	N/A	Not a measure of child maltreatment
84	Medical history questionnaire[b] (Famularo, Fenton, & Kinscherff, 1992)	N/A	Not a measure of child maltreatment
85	Minnesota Multiphasic Personality Inventory-2 (Butcher, Dahlstrom, Graham, Tellegen, & Kreammer, 1989)	MMPI-2	Not a measure of child maltreatment
86	Multidimensional Assessment of Parenting Scale (Parent & Forehand, 2017)	MAPS	Not a measure of child maltreatment
87	Multidimensional inventory for assessment of parental functioning (Reis, Orme, Barbera-Stein, & Herz, 1987)	N/A	Not a measure of child maltreatment
88	Multidimensional Neglectful Behavior Scale: Adolescent and adult recall version (Dubowitz et al., 2011)	MNBS-A	Not a parent-report measure
89	Multidimensional Neglectful Behavior Scale–Child Report (Beyazit & Ayhan, 2018)	MNBS-CR	Not a parent-report measure
90	National council on crime and delinquency indicators (Wood, 1997)	N/A	Not a parent-report measure
91	Needs-based Assessment of Parental (guardian) Support (Bolen, Lamb, & Gradante, 2002)	NAPS	Not a measure of child maltreatment
92	Neglect scale (Harrington, Zuravin, DePanfilis, Ting, & Dubowitz, 2002)	N/A	Not a parent-report measure
93	Parent cognition scale[b] (Snarr, Slep, & Grande, 2009)	N/A	Not a measure of child maltreatment
94	Parent discipline style[b] (Mezzich et al., 2007)	N/A	Not a measure of child maltreatment
95	Parent Perception Inventory (Glaser, Horne, & Myers, 1995)	PPI	Not a measure of child maltreatment
96	Parent Perception Inventory–Child version (Bruce et al., 2006)	PPIC	Not a measure of child maltreatment
97	Parent Problem Checklist (Stallman, Morawska, & Sanders, 2009)	PPC	Not a measure of child maltreatment
98	Parent Qualities Measure (Crick, 2006; Stallman et al., 2009)	PQM	Not a measure of child maltreatment
99	Parent Threat Inventory (Crick, 2006; Scher, Stein, Ingram, Malcarne, & McQuaid, 2002)	PTI	Not a parent-report measure
100	Parental Acceptance–Rejection Questionnaire (Rohner & Khaleque, 2005)	PARQ	Not a parent-report measure
101	Parental Anger Inventory (Scher et al., 2002; Sedlar & Hansen, 2001)	PAI	Not a measure of child maltreatment
102	Parental Authority Questionnaire (Buri, 1991)	PAQ	Not a measure of child maltreatment
103	Parental Emotion Regulation Inventory (Lorber, Del Vecchio, Feder, & Smith Slep, 2017; Sedlar & Hansen, 2001)	PERI	Not a measure of child maltreatment
104	Parental Empathy Measure (Kilpatrick, 2005; Lorber et al., 2017)	PEM	Not a measure of child maltreatment
105	Parent–Child Activities interview (Kilpatrick, 2005; Lefever et al., 2008)	PCA	Not a parent-report measure
106	Parent–Infant Relationship Global Assessment Scale (Lefever et al., 2008; THREE, 2005)	PIR-GAS	Not a measure of child maltreatment
107	Parenting Anxious Kids Ratings Scale–Parent Report (Flessner, Murphy, Brennan, & D'Auria, 2017; THREE, 2005)	PAKRS-PR	Not a measure of child maltreatment
108	Parenting behavior rating scales (Flessner et al., 2017; G. A. King, Rogers, Walters, & Oldershaw, 1994)	N/A	Not a parent-report measure
109	Parenting daily diary (G. A. King et al., 1994; Peterson, Tremblay, Ewigman, & Popkey, 2002)	N/A	Not a parent-report measure
110	Parenting Practices Questionnaire–Corporal Punishment (Avinun, Davidov, Mankuta, Knafo-Noam, & Knafo-Noam, 2018)	PPQ-CP	Not a measure of child maltreatment
111	Parenting Scale (Peterson et al., 2002; Salari, Terreros, & Sarkadi, 2012)	PS	Not a measure of child maltreatment

(Continued)

Appendix D. *(continued)*

No	Instrument (References)[a]	Abbreviation	Reason for exclusion
112	Parenting Support Needs Assessment (Murry & Lewin, 2014; Salari et al., 2012)	PSNA	Not a measure of child maltreatment
113	Plotkin Child Vignettes (Plotkin, 1983)	PCV	Not a measure of child maltreatment
114	Post–divorce Parental Conflict Scale (Morris & West, 2000; Murry & Lewin, 2014)	PPCS	Not a measure of child maltreatment
115	PREschool Symptom Self-report (Martini, Strayhorn, & Puig-Antich, 1990)	PRESS	Not a measure of child maltreatment
116	Production of Discipline Alternatives (Rodriguez, Wittig, & Christl, 2019)	PDA	Not a parent-report measure
117	Protective Factors Survey (Counts, Buffington, Chang-Rios, Rasmussen, & Preacher, 2010; Martini et al., 1990)	PFS	Not a measure of child maltreatment
118	Psychological Maltreatment Rating Scales (Brassard, Hart, & Hardy, 1993; Counts et al., 2010)	PMRS	Not a parent-report measure
119	Psychological neglect (Brassard et al., 1993; Christ, Kwak, & Lu, 2017)	N/A	Not a parent-report measure
120	Psychologically Violent Parental Practices Inventory (Christ et al., 2017; Gagne, Pouliot-Lapointe, & St-Louis, 2007)	PVPPI	Not developed in English
121	Questionnaire for evaluating maltreatment and neglect (Calheiros, Patrício, Graça, & Magalhães, 2018)	N/A	Not developed in English
122	Reflective Parenting Assessment (Ensink, Leroux, Normandin, Biberdzic, & Fonagy, 2017; Gagne et al., 2007)	RPA	Not a measure of child maltreatment
123	Responsiveness index (Ensink et al., 2017; Yates, Hull, & Huebner, 1983)	N/A	Not a parent-report measure
124	Revised Child Anxiety and Depression Scale Parent version (Ebesutani, Tottenham, & Chorpita, 2015; Yates et al., 1983)	RCADS-P	Not a measure of child maltreatment
125	Risk scale[b] (Ebesutani et al., 2015; Grietens, Geeraert, & Hellinckx, 2004)	N/A	Not a parent-report measure
126	Rorschach Inkblot Method (Choca, 2013; Grietens et al., 2004)	RIM	Not a measure of child maltreatment
127	Scale of Negative Family Interactions (Choca, 2013; Simonelli, Mullis, & Rohde, 2005)	SNFI	Not a parent-report measure
128	Screen for Adolescent Violence Exposure for children version (Flowers, Lanclos, & Kelley, 2002; Simonelli et al., 2005)	KID-SAVE	Not a parent-report measure
129	Sexual Abuse Indicators (Flowers et al., 2002; Terrell et al., 2008)	SAI	Not a parent-report measure
130	Sexual behavior problems questionnaire[b] (Hall, Mathews, & Pearce, 1998; Terrell et al., 2008)	N/A	Not a parent-report measure
131	Sexual Events Questionnaire (Finkelhor, 1979; Hall et al., 1998)	SEQ	Not a parent-report measure
132	Sexual Experiences Survey (Finkelhor, 1979; Koss & Gidycz, 1985)	SES	Not a parent-report measure
133	Shaken Baby Syndrome awareness assessment (Koss & Gidycz, 1985; Russell & Britner, 2006)	SBS	Old version of a revised measure
134	Sixteen Personality Factor questionnaire (Francis, Hughes, & Hitz, 1992; Russell & Britner, 2006)	16-PF	Not a measure of child maltreatment
135	Social Factors and Children Violence Questionnaire (Francis et al., 1992; Oni & Adetoro, 2014)	SPCVQ	No psychometric data found
136	Standardized Observation Codes III (Cerezo, Keesler, Dunn, & Wahler, 1986; Oni & Adetoro, 2014)	SOC III	Not a measure of child maltreatment
137	Structured Problem Analysis of Raising Kids (Cerezo et al., 1986; Staal, van den Brink, Hermanns, Schrijvers, & van Stel, 2011)	SPARK	Not a measure of child maltreatment
138	Supervisory neglect (Coohey, 2003; Staal et al., 2011)	N/A	Not a parent-report measure
139	Symptoms Of Trauma Scale (Coohey, 2003; Ford et al., 2017)	SOTS	Not a measure of child maltreatment
140	Trauma Experiences Checklist (Cristofaro et al., 2013; Ford et al., 2017)	TEC	Not a measure of child maltreatment
141	Trauma History Questionnaire (Cristofaro et al., 2013; Hooper, Stockton, Krupnick, & Green, 2011)	THQ	Not a parent-report measure
142	Trauma Symptom Checklist for Children (Briere et al., 2001; Hooper et al., 2011)	TSCC	Not a measure of child maltreatment
143	Trauma Symptom Checklist for Young Children (Briere et al., 2001)	TSCYC	Not a measure of child maltreatment
144	U.S. Air Force Family Advocacy Program Severity Index (Briere et al., 2001; A. M. Slep & Heyman, 2004)	USAF-FAP Severity Index	Not a parent-report measure

(Continued)

Appendix D. *(continued)*

No	Instrument (References)[a]	Abbreviation	Reason for exclusion
145	Violent Experiences Questionnaire–Revised (A. R. King & Russell, 2017; A. M. Slep & Heyman, 2004)	VEQ-R	Not a parent-report measure
146	Weekly Problems Scales (A. R. King & Russell, 2017; Sawyer, Tsao, Hansen, & Flood, 2006)	WPS	Not a measure of child maltreatment
147	When Bad Things Happen scale (Fletcher, 1995; Sawyer et al., 2006)	WBTH	Not a measure of child maltreatment
148	Young Parenting Inventory (Young, Klosko, & Weishaar, 2003)	YPI	Not a parent-report measure
149	Young Parenting Inventory–Revised (Louis, Wood, & Lockwood, 2018)	YPI-R2	Not a parent-report measure
150	Young Schema Questionnaire–Short form 3 (Young, 2005)	YSQ-S3	Not a parent-report measure

Notes. N/A = Not Applicable (No Abbreviation).

[a] References of the excluded instruments in this review are available from the first author upon request.

[b] Unofficial title retrieved from publication content as an instrument published without a title or abbreviation.
