# Supplementary material for: A Systematic Review Evaluating Psychometric Properties of Parent or Caregiver Report Instruments on Child Maltreatment: Part 2: Internal Consistency, Reliability, Measurement Error, Structural Validity, Hypothesis Testing, Cross-Cultural Validity, and Criterion Validity
Source: Trauma Violence Abuse. 2020 Apr 9;22(5):1296–315. doi: 10.1177/1524838020915591 (PMC8739544; doi:10.1177/1524838020915591)
Supplement: Supplemental_Material - A Systematic Review Evaluating Psychometric Properties of Parent or Caregiver Report Instruments on Child Maltreatment: Part 2: Internal Consistency, Reliability, Measurement Error, Structural Validity, Hypothesis Testing, Cross-Cultural Validity, and Criterion Validity [file Supplemental_Material.zip › Appendix E.pdf]

Appendix E. *Descriptions of Included Studies on Psychometric Properties of Instruments for the Assessment of Child Maltreatment.*

Instrument (Abbreviation)	Reference	Purpose of study	Assessed Psychometric properties	Study population	Age (Range [R] and/or Mean [MN] and/or Standard Deviation [SD])
**Adult Adolescent Parenting Inventory-2 (AAPI-2)**	Bavolek and Keene (1999)	To develop and validate the AAPI-2	Structural validity Internal consistency Hypotheses testing for construct validity	N = 1427 (Stage: Construct development): (I) Adolescents and adult parents<br>N = 989 (Stage: Validation of the AAPI-2): (II) Non-Abusive parents (F = 677; M = 225); (III) Abusive parents (F = 58; M = 29)	(I) R = NR, MN = NR, SD = NR; (II) R = NR, MN = NR, SD = NR; (III) R = NR, MN = NR, SD = NR
	Conners et al. (2006)	To examine the psychometric properties of the AAPI-2	Structural validity Internal consistency Hypotheses testing for construct validity	N = 309: Low-income parents of preschool age children (F = NR; M = NR)	R = 21–30y, MN = NR, SD = NR
	Lawson et al. (2017)	To examine the construct and predictive validity of the AAPI-2	Structural validity Internal consistency Hypotheses testing for construct validity	N = 2,610: Participating parents in child maltreatment prevention programs (F = 2,583; M = 27): (I) n = 1,271: Parents completing the AAPI-2 only before the programs (F = 1,258; M = 13); (II) n = 1,339: Parents completing the AAPI-2 both before and after the programs (F = 1,325; M = 14)	(I) R = NR, MN = NR, SD = NR; (II) R = NR, MN = NR, SD = NR
	Rodriguez et al. (2011)	To develop and validate the P-CAAM (correlation with AAPI-2, CAP, and APT)	Internal consistency Hypotheses testing for construct validity	N = 147 (Stage: Pilot Testing): (I) Undergraduate students (F = 105; M = 42)<br>N = 70 (Stage: Validation of P-CAAM by comparing with AAPI-2): (II) Mothers of children younger than 12 years old (F = 70; M = 0)	(I) R = NR, MN = 18.91y, SD = 2.35y; (II) R = NR, MN = 36.71y, SD = 6.59y
	Russa and Rodriguez (2010)	To support the validity of the APT as a questionnaire to assess risk for harsh, physically aggressive parenting (correlation with AAPI-2 and CAP)	Hypotheses testing for construct validity	N = 66 (Stage: Correlation study between APT and AAPI-2): (I) Pre-parent undergraduate students (F = 55; M = 11)<br>N = 181 (Stage: Correlation study between APT, ATS and AAPI-2): (II) Pre-parent undergraduate students (F = 134; M = 47)<br>N = 324 (Stage: Correlation study between APT, ATS and CAP): (III) Pre-parent undergraduate students (F = 220; M = 104)	(I) R = NR, MN = 18.76y, SD = 1.56y; (II) R = NR, MN = 18.91y, SD = 2.40y; (III) R = NR, MN = 19.13y, SD = 2.45y
**Analog Parenting Task (APT)**	Rodriguez et al. (2011)	To develop and validate the P-CAAM (correlation with AAPI-2, CAP, and APT)	Internal consistency Hypotheses testing for construct validity	N = 147 (Stage: Pilot Testing): (I) Undergraduate students (F = 105; M = 42)<br>N = 70 (Stage: Validation of P-CAAM by comparing with AAPI-2): (II) Mothers of children younger than 12 years old (F = 70; M = 0)	(I) R = NR, MN = 18.91y, SD = 2.35y; (II): R = NR, MN = 36.71y, SD = 6.59y
	Russa and Rodriguez (2010)	To support the validity of the APT as an questionnaire to assess risk for harsh, physically aggressive parenting (correlation with AAPI-2, ATS, and CAP)	Internal consistency Hypotheses testing for construct validity	N = 66 (Stage: Correlation study between APT and AAPI-2): (I) Pre-parent undergraduate students (F = 55; M = 11)<br>N = 181 (Stage: Correlation study between APT, ATS and AAPI-2): (II) Pre-parent undergraduate students (F = 134; M = 47)<br>N = 324 (Stage: Correlation study between APT, ATS and CAP): (III) Pre-parent undergraduate students (F = 220; M = 104)	(I) R = NR, MN = 18.76y, SD = 1.56y; (II) R = NR, MN = 18.91y, SD = 2.40y; (III) R = NR, MN = 19.13y, SD = 2.45y

(Continued)

Appendix E. *(continued)*

Instrument (Abbreviation)	Reference	Purpose of study	Assessed Psychometric properties	Study population	Age (Range [R] and/or Mean [MN] and/or Standard Deviation [SD])
**Child Neglect Questionnaire (CNQ)**	Kirisci et al. (2001)	To develop and evaluate psychometric properties of the CNQ	Structural validity Internal consistency Hypotheses testing for construct validity	N = 172: (I) n = 76: Parents of children having fathers with Substance Use Disorder (SUD); (II) n = 96: Parents of children having fathers without SUD	(I) R = NR, MN = NR, SD = NR; (II) R = NR, MN = NR, SD = NR
**Child Neglect Scales–Maternal Monitoring and Supervision Scale (CNS-MMS)**	Stewart et al. (2015)	To develop and evaluate validity and reliability of the Child Neglect Scales (CNS)	Structural validity Internal consistency Hypotheses testing for construct validity	N = 344: (I) n = 122: Mothers of boys having fathers with Substance Use Disorder (SUD) (F = 122; M = 0); (II) n = 222: Mothers of boys having fathers without SUD (F = 222; M = 0)	(I) R = NR, MN = NR, SD = NR; (II) R = NR, MN = NR, SD = NR
**Child Trauma Screen–Exposure Score (CTS-ES)**	Lang and Connell (2017)	To develop and validate the Child Trauma Screen (CTS)	Hypotheses testing for construct validity	N = 923 (Stage: CTS Development): (I) Parents of children receiving care at outpatient behavioral health clinics<br>N = 69 (Stage: CTS Validation): (II) Parents of children receiving care at outpatient behavioral health clinics	(I) R = NR, MN = NR, SD = NR; (II) R = NR, MN = NR, SD = NR
**Conflict Tactics Scales: Parent–Child version (CTSPC)**	Compier-de Block et al. (2017)	To examine to what extent parents and children agree on the occurrence of various types of parent-to-child maltreatment by comparing parent and child report CTSPC	Internal consistency Reliability Hypotheses testing for construct validity	N = 83: Parents reported on perpetrated maltreatment (F = 48; M = 35)	R = 33–88y, MN = 57.4y, SD = 11.5y
	Cotter et al. (2018)	To examine the factor structure of the CTSPC as well as its convergent validity with the DPICS	Structural validity Internal consistency Hypotheses testing for construct validity	N = 110: Parents with a substantiated physical abuse incident who were court-mandated to receive treatment (F = 72; M = 38)	R = NR, MN = 32.24y, SD = 8.68y
	Grasso et al. (2016)	To examine the overlap between specific forms of psychological and physical Intimate partner violence by using the CTS-2 and mothers' harsh parenting behaviors by using the CTSPC, and their relationship to child disruptive behavior by using the MAP-DB	Internal consistency	N = 162: (I) n = 81: Mothers of children ages 4 to 6 years (F = 81; M = 0); (II) n = 81: Children ages 4 to 6 years (F = 31; M = 50)	(I) R = NR, MN = 31.1y, SD = 5.4y; (II) R = 4–6y, MN = 4.74y, SD = 0.91y
	Kobulsky et al. (2017)	To investigate the concordance of parent and child reports of current physical abuse by using the CTSPC, and the relation between concordance and parent and child reports of current child behavioral problems by using the CBCL and the YSR.	Reliability	N = 1,376: (I) n = 638: Parents reported on child physical abuse (F = 572; M = 66); (II) n = 638: Children reported on child physical abuse (F = 369; M = 269)	(I) R = 22–87y, MN = 39.5y, SD = 8.4y; (II) R = 11–17y, MN = 13.6y, SD = 1.9y

(Continued)

Appendix E. *(continued)*

Instrument (Abbreviation)	Reference	Purpose of study	Assessed Psychometric properties	Study population	Age (Range [R] and/or Mean [MN] and/or Standard Deviation [SD])
**Conflict Tactics Scales: Parent–Child version (CTSPC)**	Lorber and Slep (2017)	To prove the reliability of CTSPC by using Item Response Theory (IRT) analyses	Structural validity<br>Internal consistency	N = 453: parents with 3- to 7-year old children (F = 235; M = 218)	R = NR, MN = NR, SD = NR
	O'Dor et al. (2017)	To exam the psychometric properties of the FSI-R (correlation with CTSPC)	Internal consistency<br>Hypotheses testing for construct validity	N = 772: (I) n = 386: Mothers of 3–6 year-old children with disruptive behavior or experience of intimate partner violence (IPV); (II) n = 386: 3–6 year-old children with disruptive behavior or parents exposed intimate partner violence (IPV)	(I) R = NR, MN = NR, SD = NR; (II) R = NR, MN = 56.72m, SD = 10.27m
	Rodriguez (2010)	To explore relationships between parent–child aggression by using the CTSPC and parenting styles by using the PS associated with child maltreatment potential by using the CAP	Hypotheses testing for construct validity	N = 772: (I) n = 327: Parents of children younger than 12 (F = 275; M = 52); (II) n = 115: parents of children between ages 7 and 12 (F = 86; M = 29); (III) n = 74: Mothers of 7- to 12-year-old children with diagnosed externalising behavior problems (F = 74; M = 0)	(I) R = NR, MN = 30.48y, SD = 6.22y; (II) R = NR, MN = 37.62y, SD = 7.91y; (III) R = NR, MN = 40.65y, SD = 10.53y
	Straus et al. (1998)	To develop and test the reliability and validity of CTSPC	Internal consistency<br>Hypotheses testing for construct validity	N = 1,000: Parents of children under 18 years old participated in an U.S. national survey (F = 660; M = 340)	R = NR, MN = 36.8y, SD = NR
**Family Maltreatment–Child Abuse criteria (FM-CA)**	Heyman, et al. (2019).	To develop and validate the FM-CA	Hypotheses testing for construct validity	N = 126: U.S. Air Force service members and their spouses (F = 41; M = 85)	R = NR, MN = NR, SD = NR
**ISPCAN Child Abuse Screening Tool for use in Trials (ICAST-Trial)**	Meinck et al. (2018)	To develop and validate the ICAST-Trial	Structural validity<br>Internal consistency<br>Hypotheses testing for construct validity	N = 115 (Stage: Pilot study) (I) Parents of adolescents participated in a parenting program to prevent child abuse (F = 112; M = 3)<br>N = 552 (Stage: Validation of ICAST-Trial) (II) Parents of adolescents participated in a parenting program to prevent child abuse (F = 523; M = 29)	(I) R = NR, MN = 48y, SD = 13.6y; (II) R = NR, MN = 49.4y, SD = 14.69y
**Intensity of Parental Punishment Scale (IPPS)**	Gordon et al. (1979)	To develop and validate the IPPS	Structural validity<br>Internal consistency<br>Cross-cultural validity<br>Reliability<br>Hypotheses testing for construct validity	N = 417: (I) n = 301: Parents of 5- to 10-year-old children; (II) n = 50: Upper-middle-class parents of 7- to 12-year old children; (III) n = 26: Mothers of 6- to 9-year-old children; (IV) n = 40: Mothers of 6- to 14-year-old children	(I) R = NR, MN = NR, SD = NR; (II) R = NR, MN = NR, SD = NR; (III) R = NR, MN = NR, SD = NR; (IV) R = NR, MN = NR, SD = NR
**Mother–Child Neglect Scale (MCNS)**	Lounds et al. (2004)	To evaluate reliability and validity of the MCNS and MCNS-SF	Internal consistency<br>Reliability<br>Hypotheses testing for construct validity	N = 100: Adolescent mothers of children ages 9 to 15 years	R = 14.2–19.2y, MN = 17y, SD = 1.16y

(Continued)

Appendix E. *(continued)*

Instrument (Abbreviation)	Reference	Purpose of study	Assessed Psychometric properties	Study population	Age (Range [R] and/or Mean [MN] and/or Standard Deviation [SD])
**Mother–Child Neglect Scale–Short Form (MCNS-SF)**	Lounds et al. (2004)	To evaluate reliability and validity of the MCNS and MCNS-SF	Internal consistency<br>Criterion validity<br>Hypotheses testing for construct validity	N = 100: Adolescent mothers of children ages 9 to 15 years	R = 14.2–19.2y, MN = 17y, SD = 1.16y
**Parent–Child Aggression Acceptability Movie task (P-CAAM)**	Rodriguez et al. (2011)	To develop and validate the P-CAAM (correlation with AAPI-2, CAP, and APT)	Internal consistency<br>Hypotheses testing for construct validity	N = 147 (Stage: Pilot Testing): (I) Undergraduate students (F = 105; M = 42)<br>N = 70 (Stage: Validation of P-CAAM by comparing with AAPI-2): (II) Mothers of children younger than 12 years old (F = 70; M = 0)	(I) R = NR, MN = 18.91y, SD = 2.35y; (II): R = NR, MN = 36.71y, SD = 6.59y
**Parent Opinion Questionnaire (POQ)**	Azar and Rohrbeck (1986)	To assess validation of the POQ by comparing the unrealistic expectations of child abusing mothers with mothers whose partners perpetrated the abuse	Reliability<br>Hypotheses testing for construct validity	N = 30: (I) n = 16 Mothers abusing their children; (II) n = 14: Non-abusing mothers with partners abusing their children	(I) R = NR, MN = NR, SD = NR; (II) R = NR, MN = NR, SD = NR
	Haskett et al. (2006)	To exam psychometric properties of the POQ and CV	Structural validity<br>Internal consistency<br>Hypotheses testing for construct validity	N = 155: (I) n = 77: Abusive parents documented history of child physical abuse with 4- to 10-year-old children (F = 64; M = 13); (II) n = 78: Non-abusive parents with 4- to 10-year-old children (F = 64; M = 14)	(I) R = NR, MN = 34.3y, SD = 7.2y; (II) R = NR, MN = 34.7y, SD = 9.3y
	Mammen et al. (2003)	To exam convergence among cognitions by using the POQ and satisfaction with the child by using the CRI in child abusive parents, and their relationships to parental aggression by using the CTS	Hypotheses testing for construct validity	N = 52: Parent participants in a treatment study because of physical abuse towards their children ages 6 to 13 years (F = 44; M = 8)	R = NR, MN = 31.9y, SD = 5.88y
**Parental Response to Child Misbehavior questionnaire (PRCM)**	Vittrup et al. (2006)	To exam the emergence of discipline techniques by mothers of young children by using the PRCM and assess the predictive validity of spanking attitudes with subsequent reports of spanking by using the ATS	Hypotheses testing for construct validity	N = 132: Mothers of 12- to 48-month-old children (F = 132; M = 0)	R = 20–44y, MN = 31.4y, SD = 4.5y
**Shaken Baby Syndrome awareness assessment–Short Version (SBS-SV)**	Russell (2010)	To develop and assess the psychometric properties of the SBS-SV	Internal consistency	N = 370: Public college students (F = 270; M = 100)	R = NR, MN = 21y, SD = NR

Note. AEIII = Assessing Environments III; ATS = Attitude Towards Spanking; CAP = Child Abuse Potential inventory; CBCL = Child Behavior CheckList; CRI = Child Rearing Inventory; CTS = Conflict Tactics Scale; CTS-2 = Conflict Tactics Scale-2; CV = Child Vignettes; DPICS = Dyadic Parent–child Interaction Coding System; FSI-R = Family Socialization Interview–Revised; ISPCAN = International Society for the Prevention of Child Abuse and Neglect; MAP-DB = Multidimensional Assessment of Preschool Disruptive Behavior; PS = Parenting Scale; YSR = Youth Self-Report.
