# Supplementary material for: A Systematic Review Evaluating Psychometric Properties of Parent or Caregiver Report Instruments on Child Maltreatment: Part 2: Internal Consistency, Reliability, Measurement Error, Structural Validity, Hypothesis Testing, Cross-Cultural Validity, and Criterion Validity
Source: Trauma Violence Abuse. 2020 Apr 9;22(5):1296–315. doi: 10.1177/1524838020915591 (PMC8739544; doi:10.1177/1524838020915591)
Supplement: Supplemental_Material - A Systematic Review Evaluating Psychometric Properties of Parent or Caregiver Report Instruments on Child Maltreatment: Part 2: Internal Consistency, Reliability, Measurement Error, Structural Validity, Hypothesis Testing, Cross-Cultural Validity, and Criterion Validity [file Supplemental_Material.zip › Appendix F.pdf]

Appendix F. *Study Results and Ratings on Psychometric Properties.*

Psychometric Property	Instrument	Reference	Risk of bias	Sample size	Results (rating)	Overall rating	Quality of evidence (reasons)
**Structural validity**	**AAPI-2**	Bavolek and Keene (1999)	Very good	1,427	*EFA*: 5-factor structure **(?)**	±	**Moderate** (partly inconsistent results)
		Conners et al. (2006)	Very good	309	*CFA*: 5-factor structure: 1st factor CFI = 0.86; 2nd factor = 0.68; 3rd factor = 0.82; 4th factor = 0.75; 5th factor = 1.0 **(-)**		
		Lawson et al. (2017)	Adequate	1,271	*CFA*: 5-factor structure: CFI = 0.84; TLI = 0.84; RMSEA = 0.14 **(-)**		
			Adequate	1,339	*CFA*: 2-factor structure: CFI = 0.901; TLI = 0.968; RMSEA = 0.058 **(+)**		
	**CNQ**	Stewart et al. (2015)	Adequate	172	*CFA*: 1-factor structure: Total scale and each subscale RMSEA < 0.001 **(+)**	+	**Moderate** (only one adequate study)
	**CNS-MMS**	Kirisci et al. (2001)	Very good	344	*CFA*: 1-factor structure: RMSEA = 0.028 **(+)**	+	**High** (no concern)
	**CTSPC**	Cotter et al. (2018)	Very good	110	*EFA:* 4-factor structure **(?)**	?	**Not evaluated** (lack of evidence)
		Lorber and Slep (2017)	Very good	453	*CFA:* Physical assault subscale CFI > 0.95; Total scale and other subscales: NR **(?)**		
			Very good	453	IRT: Physical assault subscale CFI > 0.95; Evidence of Local Independence: NR; Adequate looking graphs for Monotonicity: NR; x^2 > 0.01; Total scale and other subscales: NR **(?)**		
	**ICAST-Trial**	Meinck et al. (2018)	Very good	552	CFA: 4-factor structure: CFI 0.975, TLI 0.965, RMSEA 0.025, SRMR 0.036 **(+)**	+	**High** (no concern)
	**IPPS**	Gordon et al. (1979)	Adequate	217	*EFA*: 5-factor structure **(?)**	?	**Not evaluated** (lack of evidence)
	**POQ**	Haskett et al. (2006)	Doubtful	128	*EFA*: 6-factor structure **(?)**	?	**Not evaluated** (lack of evidence)
**Internal consistency**	**AAPI-2**	Bavolek and Keene (1999)	Very good	1,427	Conflicting structural validity *AND* Cronbach's Alpha = 0.82 (1st factor); 0.88 (2nd factor); 0.92 (3rd factor); 0.82 (4th factor); 0.80 (5th factor) **(?)**	?	**Not evaluated** (lack of evidence)
		Conners et al. (2006)	Very good	309	Conflicting structural validity *AND* Cronbach's alpha = 0.85 (Total scale); 0.79 (1st factor); 0.64 (2nd factor); 0.79 (3rd factor); 0.59 (4th factor); 0.50 (5th factor) **(?)**		
		Lawson et al. (2017)	Very good	1,271	Conflicting structural validity *AND* Cronbach's Alpha = 0 .89 (Total scale); 0.70 (1st factor); 0.69 (2nd factor); 0.70 (3rd factor); 0.56 (4th factor); 0.48 (5th factor) **(?)**		
		Rodriguez et al. (2011)	Adequate	147	Conflicting structural validity *AND* Cronbach's Alpha = 0.87 **(?)**		
			Very good	70	Conflicting structural validity *AND* Cronbach's Alpha = 0.83 **(?)**		
	**APT**	Rodriguez et al. (2011)	Very good	147	No evidence on the structural validity AND Cronbach's Alpha = 0.92 (Physical Discipline); 0.72 (Escalation) **(?)**	?	**Not evaluated** (lack of evidence)
		Russa and Rodriguez (2010)	Very good	66	No evidence on the structural validity AND Cronbach's Alpha = 0.93 (Physical Discipline); 0.80 (Escalation) **(?)**		
			Very good	181	No evidence on the structural validity AND Cronbach's Alpha = 0.91 (Physical Discipline); 0.77 (Escalation) **(?)**		
			Very good	324	No evidence on the structural validity AND Cronbach's Alpha = 0.92 (Physical Discipline); 0.72 (Escalation) **(?)**		

(Continued)

Appendix F. *(Continued).*

Psychometric Property	Instrument	Reference	Risk of bias	Sample size	Results (rating)	Overall rating	Quality of evidence (reasons)
**Internal consistency**	**CNQ**	Stewart et al. (2015)	Doubtful	172	Moderate evidence for sufficient structural validity *AND* Cronbach's Alpha = 0.86 (mother report); 0.92 (father report) **(+)**	**+**	**Low** (only one doubtful study)
	**CNS-MMS**	Kirisci et al. (2001)	Very good	344	High evidence for sufficient structural validity *AND* Cronbach's Alpha = 0.72 **(+)**	**+**	**High** (no concern)
	**CTSPC**	Compier-de Block et al. (2017)	Very good	35	Indeterminate structural validity *AND* Cronbach's Alpha (Father report) = 0.74 (Total scale); 0.64 (Psychological Aggression); 0.71 (Physical Assault); 0.78 (Emotional Neglect) **(?)**	**?**	**Not evaluated** (lack of evidence)
			Very good	48	Indeterminate structural validity *AND* Cronbach's Alpha (Mother report) = 0.82 (Total scale); 0.75 (Psychological Aggression); 0.68 (Physical Assault); 0.79 (Emotional Neglect) **(?)**		
		Cotter et al. (2018)	Adequae	110	Indeterminate structural validity *AND* Cronbach Alpha = 0.72 (Corporal Punishment/Minor Physical Assault); 0.75 (Psychological Aggression scale); 0.72 (Nonviolent Discipline scale); 0.46 (Severe Physical Assault) **(?)**		
		Grasso et al. (2016)	Very good	81	Indeterminate structural validity *AND* Cronbach's Alpha = 0.61 (Psychological Aggression); 0.59 (Mild/Moderate Child Physical Assault); 0.54 (Severe Child Physical Assault) **(?)**		
		Lorber and Slep (2017)	Adequate	453	Indeterminate structural validity *AND* Cronbach's Alpha ≤ 0.59 **(?)**		
		O'Dor et al. (2017)	Very good	386	Indeterminate structural validity *AND* Cronbach's Alpha = 0.65 (Physical Aggression); 0.68 (Psychological Aggression) **(?)**		
		Straus et al. (1998)	Adequate	1,000	Indeterminate structural validity *AND* Cronbach's Alpha = 0.55 (Physical Assault); 0.60 (Psychological Aggression); 0.70 (Nonviolent Discipline); 0.22 (Neglect) **(?)**		
	**ICAST-Trial**	Meinck et al. (2018)	Very good	552	High-quality evidence for sufficient structural validity *AND* Cronbach's Alpha = 0.84 (1st factor); 0.62 (2nd factor); 0.62 (3rd factor); 0.58 (4th factor) **(-)**	**-**	**High** (no concern)
	**IPPS**	Gordon et al. (1979)	Very good	217	Indeterminate structural validity *AND* Spilt-half reliability coefficient (r = 0.75, p < 0.01) **(?)**	**?**	**Not evaluated** (lack of evidence)
	**MCNS**	Lounds et al. (2004)	Very good	100	No evidence on the structural validity *AND* Coefficient Alpha = 0.94 (Total Scale); 0.80 (Emotional Needs); 0.86 (Cognitive Needs); 0.78 (Supervision); 0.90 (Physical Neglect) **(?)**	**?**	**Not evaluated** (lack of evidence)
			Very good	100	No evidence on the structural validity *AND* Coefficient Alpha = 0.95 (Total Scale); 0.85 (Emotional Needs); 0.86 (Cognitive Needs); 0.86 (Supervision); 0.91 (Physical Neglect) **(?)**		
	**MCNS-SF**	Lounds et al. (2004)	Very good	100	No evidence on the structural validity *AND* Coefficient Alpha = 0.90 **(?)**	**?**	**Not evaluated** (lack of evidence)
	**P-CAAM**	Rodriguez et al. (2011)	Adequate	147	No evidence on the structural validity *AND* Alpha = 0.77 (university student sample) **(?)**	**?**	**Not evaluated** (lack of evidence)
			Very good	70	No evidence on the structural validity *AND* Alpha = 0.74 (parent sample) **(?)**		
	**POQ**	Haskett et al. (2006)	Very good	128	Indeterminate structural validity *AND* KR-20 = 0.82 (Total scale); 0.54 (Self Care); 0.45 (Family Responsibility); 0.56 (Help/Affection to Parents); 0.31 (Leave Child Alone); 0.53 (Proper Behavior Feelings); 0.47 (Punishment) **(?)**	**?**	**Not evaluated** (lack of evidence)
	**SBS-SV**	Russell (2010)	Very good	370	No evidence on the structural validity *AND* Cronbach's Alpha = 0.76 (Soothing Technique); 0.79 (Discipline Techniques); 0.70 (Potential for Injury) **(?)**	**?**	**Not evaluated** (lack of evidence)

(Continued)

Appendix F. *(Continued).*

Psychometric Property	Instrument	Reference	Risk of bias	Sample size	Results (rating)	Overall rating	Quality of evidence (reasons)
**Cross-cultural validity**	**IPPS**	Gordon et al. (1979)	Inadequate	217	Multiple group factor analysis or DIF: NR **(?)**	**?**	**Not evaluated** (lack of evidence)
**Reliability**	**CTSPC**	Compier-de Block et al. (2017)	Very good	35	Interrater reliability: ICC = 0.29 **(-)**	**-**	**Moderate** (some indirect evidence from different population other than target population)
			Very good	48	Interrater reliability: ICC = 0.18 **(-)**		
		Kobulsky et al. (2017)	Very good	638	Interrater reliability: Kappa = 0.144 **(?)**		
	**IPPS**	Gordon et al. (1979)	Doubtful	19	Test-retest reliability: r = 0.85; statistical method not reported **(?)**	**?**	**Not evaluated** (lack of evidence)
			Doubtful	50	Test-retest reliability: r = 0.56; statistical method not reported **(?)**		
	**MCNS**	Lounds et al. (2004)	Adequate	100	Test-retest reliability: Spearman's rho = 0.60 **(?)**	**?**	**Not evaluated** (lack of evidence)
	**POQ**	Azar and Rohrbeck (1986)	Doubtful	16	Test-retest reliability: r = 0.85; statistical method not reported **(?)**	**?**	**Not evaluated** (lack of evidence)
**Criterion validity**	**MCNS-SF**	Lounds et al. (2004)	Very good	100	Correlation with MCNS (long version): r = 0.96 **(+)**	**+**	**High** (no concern)
**Hypotheses testing for construct validity**	**AAPI-2**	Bavolek and Keene (1999)	Adequate	989	Difference between abusive and non-abusive parents: Cohen's d = 0.57–3.96 **(+)**	**-**	**Moderate** (some indirect evidence from different population other than target population)
			Adequate	989	Difference between fathers and mothers: Cohen's d = 0.28–1.40 **(-)**		
		Conners et al. (2006)	Adequate	309	Correlation with PDMI: r = -0.36 **(-)**		
			Very good	309	Correlation with HOME r = 0.19 **(-)**		
			Very good	309	Correlation with Parenting Style: r = -0.45 **(-)**		
			Very good	309	Correlation with PKBS: r = -0.23 **(-)**		
		Lawson et al. (2017)	Adequate	1,339	Difference between parents of children with a substantiated child maltreatment report (SCAR) and without SCAR (2-factor AAPI-2): Cohen's d = 0.04 **(-)**		
			Adequate	1,339	Difference between parents of children with a substantiated child maltreatment report (SCAR) and without SCAR (5-factor AAPI-2): Cohen's d = 0.03–0.11 **(-)**		
		Rodriguez et al. (2011)	Very good	147	Correlation with P-CAAM: r = -0.33 **(-)**		
			Very good	70	Correlation with P-CAAM: r = -0.51 **(+)**		
		Russa and Rodriguez (2010)	Very good	66	Correlation with APT: r = 0.353 **(-)**		
			Very good	181	Correlation with APT: r = 0.497 **(-)**		
	**APT**	Rodriguez et al. (2011)	Very good	147	Correlation with P-CAAM: r = 0.26–0.29 **(-)**	**±**	**Very Low** (partly inconsistent results, all indirect evidence from different population other than target population)
		Russa and Rodriguez (2010)	Very good	66	Correlation with AAPI-2: r = 0.339–0.353 **(-)**		
			Very good	181	Correlation with AAPI-2: r = 0.463–0.497 **(-)**		
			Very good	324	Correlation with CAP: r = 0.158–0.279 **(-)**		
			Adequate	324	Correlation with ATS: r = 0.521–0.565 **(+)**		
			Adequate	181	Correlation with ATS: r = 0.577–0.612 **(+)**		

(Continued)

Appendix F. *(Continued).*

Psychometric Property	Instrument	Reference	Risk of bias	Sample size	Results (rating)	Overall rating	Quality of evidence (reasons)
**Hypotheses testing for construct validity**	**CNQ**	Stewart et al. (2015)	Very good	172	Correlation with CRPB: r = 0.01–0.19 **(-)**	**-**	**High** (no concern)
			Very good	172	Correlation with FAM: r = 0.00–0.13 **(-)**		
			Very good	172	Correlation with ACQ: r = 0.02–0.11 **(-)**		
			Very good	172	Correlation with CRC: r = -0.13–0.27 **(-)**		
			Very good	172	Difference between families of fathers with and without substance use disorder: Lack of information to calculate Cohen's d **(?)**		
	**CNS-MMS**	Kirisci et al. (2001)	Very good	344	Correlation with child-report CNS: r = -0.10 (boys of fathers with substance use disorder (SUD); r = -0.18 (without SUD) **(-)**	**-**	**Moderate** (some indirect evidence from different population other than target population)
	**CTS-ES**	Lang and Connell (2017)	Very good	69	Correlation with CPSS: r = 0.49 **(-)**	**±**	**Low** (totally inconsistent results)
			Very good	69	Correlation with CPSS: r = 0.71 **(+)**		
	**CTSPC**	Compier-de Block et al. (2017)	Adequate	83	Difference between parents with younger children and parents with older children: Cohen's d = 0.54 **(+)**	**-**	**High** (no concern)
		Cotter et al. (2018)	Very good	110	Correlation with DPICS = -0.21–0.26 **(-)**		
		O'Dor et al. (2017)	Very good	386	Correlation with FSI-R: r = 0.31–0.63 **(-)**		
		Rodriguez (2010)	Very good	327	Correlation with CAPI: r = -0.01–0.39 **(-)**		
			Very good	327	Correlation with PS: r = -0.08–0.56 **(-)**		
			Very good	115	Correlation with CAPI: r = 0.08–0.33 **(-)**		
			Very good	115	Correlation with PS: r = -0.03–0.56 **(-)**		
			Very good	74	Correlation with CAPI: r = -0.14–0.33 **(-)**		
			Very good	74	Correlation with PS: r = -0.27–0.48 **(-)**		
		Straus et al. (1998)	Adequate	1,000	Difference between younger and older parents: Cohen's d = -0.70–-0.24 **(-)**		
			Adequate	1,000	Difference between parents with younger and older children: Cohen's d = -0.72–-0.12 **(-)**		
			Adequate	182	Difference between European American and African Hispanic American parents: Cohen's d = 0.68 **(+)**		
			Adequate	1,000	Difference between mothers and fathers: Cohen's d = 0.1 **(-)**		
	**FM-CA**	Heyman, et al. (2019).	Doubtful	126	Correlation with CTSPC: Guilford G (inter-rater agreement coefficient) = -0.06–0.94 **(?)**	**?**	**Not evaluated** (lack of evidence)
	**ICAST-Trial**	Meinck et al. (2018)	Very good	552	Correlation with Corporal Punishment items of APQ: r = 0.457 **(-)**	**-**	**High** (no concern)

(Continued)

Appendix F. *(Continued).*

Psychometric Property	Instrument	Reference	Risk of bias	Sample size	Results (rating)	Overall rating	Quality of evidence (reasons)
**Hypotheses testing for construct validity**	**IPPS**	Gordon et al. (1979)	Adequate	42	Correlation with Intensity of Anger: r = 0.84 **(+)**	±	**Low** (partly inconsistent results, multiple doubtful studies)
			Adequate	26	Correlation with Parent–Child Interaction Code: r = -0.57–0.44 **(-)**		
			Adequate	50	Correlation with Parent's Priorities of Child Behaviors: r = 0.49 **(-)**		
			Adequate	40	Difference between mothers with less and more warmth to children: Lack of information to calculate Cohen's d **(?)**		
			Adequate	64	Difference between parents of children with and without behavior problems: Lack of information to calculate Cohen's d **(?)**		
			Doubtful	192	Correlation with Frustration Tolerance): r = 0.18 **(-)**		
			Doubtful	49	Correlation with children's personality questionnaire: -0.28–0.32 **(-)**		
			Doubtful	43	Correlation with Child Behavior Rating: r = -0.57–0.58 **(+)**		
			Doubtful	205	Difference between parents with older and younger children: Cohen's d = 0.22–0.72 **(-)**		
			Doubtful	40	Difference between more and less out of contact mothers: Lack of information to calculate Cohen's d **(?)**		
			Doubtful	40	Difference between mothers giving more and less critical evaluations: Lack of information to calculate Cohen's d **(?)**		
			Doubtful	217	Difference between parents with lower and higher socioeconomic status: Cohen's d = 0.49 **(-)**		
			Doubtful	217	Difference between parents with less and more education: Cohen's d = 0.68 **(+)**		
	**MCNS**	Lounds et al. (2004)	Very good	100	Correlation with MIS: r = -0.31 **(-)**	-	**High** (no concern)
			Very good	100	Correlation with CAP: r = 0.16 **(-)**		
			Very good	100	Correlation with NS: r = 0.32–0.36 **(-)**		
	**MCNS-SF**	Lounds et al. (2004)	Very good	100	Correlation with CAP: r = 0.19 **(-)**	-	**High** (no concern)
			Very good	100	Correlation with NS: r = 0.28 **(-)**		
			Very good	100	Correlation with MIS: r = -0.26 **(-)**		
	**P-CAAM**	Rodriguez et al. (2011)	Very good	147	Correlation with ATS: r = 0.33–0.43 **(-)**	±	**Low** (partly inconsistent results, some indirect evidence from different population other than target population)
			Very good	147	Correlation with AAPI-2: r = -0.33–-0.27 **(-)**		
			Very good	147	Correlation with APT: r = -0.21–0.30 **(-)**		
			Very good	70	Correlation with ATS: r = 0.26–0.30 **(-)**		
			Very good	70	Correlation with AAPI-2: r = -0.51–-0.46 **(-)**		
			Very good	70	Correlation with CAPI: r = 0.27–0.30 **(-)**		
			Very good	70	Correlation with Parenting Scale: r = -0.28–0.34 **(-)**		
			Very good	34	Difference between parents with higher and lower scores of CAPI: Cohen's d = 0.70–0.76 **(+)**		
			Very good	34	Difference between parents with higher and lower scores of AAPI-2: Cohen's d = 1.32–1.44 **(+)**		
			Very good	34	Difference between parents with higher and lower scores of Overreactivity: Cohen's d = 0.57–0.89 **(+)**		
			Adequate	74	Difference between parents with higher and lower scores of AAPI-2: Cohen's d = 0.92–1.11 **(+)**		

(Continued)

Appendix F. *(Continued).*

Psychometric Property	Instrument	Reference	Risk of bias	Sample size	Results (rating)	Overall rating	Quality of evidence (reasons)
**Hypotheses testing for construct validity**	**POQ**	Azar and Rohrbeck (1986)	Very good	30	Difference between mothers who perpetrated child maltreatment and whose partners perpetrated child maltreatment: Cohen's d = 2.01 **(+)**	**-**	**High** (no concern)
		Haskett et al. (2006)	Very good	128	Correlation with CV: r = 0.33–0.43 **(-)**		
			Very good	128	Correlation with PSI: r = 0.31 **(-)**		
			Very good	128	Correlation with Parent–Child Interactions: r = -0.11–0.08 **(-)**		
			Very good	128	Correlation with K-BIT: r = -0.35 **(-)**		
			Very good	128	Difference between abusive and non-abusive parents: Cohen's d = 0.24 **(-)**		
			Adequate	128	Correlation with ECBI: r = 0.06–0.09 **(-)**		
			Adequate	128	Correlation with SCL-90-R: r = 0.30 **(-)**		
			Adequate	128	Correlation with CTS: r = 0.28 **(-)**		
		Mammen et al. (2003)	Very good	48	Correlation with CRI: r = 0.02 **(-)**		
			Very good	43	Correlation with CRI: r = 0.05 **(-)**		
			Very good	39	Correlation with PAT: r = 0.04 **(-)**		
			Very good	42	Correlation with PAT: r = -0.30 **(-)**		
			Very good	39	Correlation with PPQ: r = 0.08 **(-)**		
			Very good	40	Correlation with PPQ: r = 0.09 **(-)**		
			Very good	46	Correlation with CTSPC: r = -0.23–-0.02 **(-)**		
			Very good	42	Correlation with CTSPC: r = 0.10 (Minor Violence score); r = 0.14 **(-)**		
			Doubtful	49	Difference between abusive and non-abusive parents: Cohen's d = 0.08 **(-)**		
			Doubtful	43	Difference between abusive and non-abusive parents: Cohen's d = 0.02 **(-)**		
	**PRCM**	Vittrup et al. (2006)	Very good	244	Difference between mothers of 12-month-old and 48-month-old babies: Cohen's d = 1.79 **(+)**	**+**	**High** (no concern)

AAPI-2: Adult Adolescent Parenting Inventory-2; ACQ: Areas of Change Questionnaire; APQ: Alabama Parenting Questionnaire; APT: Analog Parenting Task; APT: Attitudes Toward Spanking; CAP: Child Abuse Potential inventory; CNQ: Child Neglect Questionnaire; CNS-MMS: Child Neglect Scales–Maternal Monitoring and Supervision scale; CPSS: Child Posttraumatic Stress Scale; CRC: Child's Relationship with Caretaker; CRI: Child Rearing Inventory; CRPB: Child Report on Parental Behavior; CTS: Conflict Tactics Scale; CTS-ES: Child Trauma Screen–Exposure Score; CTSPC: Conflict Tactics Scales: Parent–Child version; CV: Child Vignettes; DPICS: Dyadic Parent–child Interaction Coding System; ECBI: Eyberg Child Behavior Inventory; FAM :Family Assessment Measure; FM-CA: Family Maltreatment–Child Abuse criteria; FSI-R: Family Socialization Interview–Revised; HOME: Home Observations for the Measurement of the Environment; ICAST-Trial: ISPCAN (International Society for the Prevention of Child Abuse and Neglect) Child Abuse Screening Tool for use in Trials; IPPS: Intensity of Parental Punishment Scale; K-BIT: Kaufman Brief Intelligence Test; MCNS: Mother–Child Neglect Scale; MCNS-SF: Mother–Child Neglect Scale–Short Form; MIS: Maternal Interaction Scale; NS: Neglect Scale; PAT: Parent Attribution Test; P-CAAM: Parent–Child Aggression Acceptability Movie task; PDMI: Parental Discipline Methods Interview; PKBS: Preschool and Kindergarten Behavior Scales; POQ : Parent Opinion Questionnaire; PPQ: Parent Practices Questionnaire; PRCM : Parental Response to Child Misbehavior questionnaire; PS: Parenting Scale; PSI-SF: Parenting Stress Index–Short Form; SBS-SV: Shaken Baby Syndrome awareness assessment–Short Version; SCL-90-R: Symptom CheckList 90–Revised; CFA: Confirmatory Factor Analysis; CFI: Comparative Fit Index; CTT: Classical Test Theory; EFA: Exploratory Factor Analysis; IRT: Item Response Theory; NR: Not Reported; RMSEA: Root Mean Square Error of Approximation; TLI: Tucker–Lewis Index; DIF: Differential Item Functioning; ICC: Intraclass Correlation Coefficient; + = Sufficient rating; ? = Indeterminate rating; - = Insufficient rating; ± = Inconsistent rating; High = High level of confidence in overall ratings; Moderate = Moderate level of confidence in overall ratings; Low = Low level of confidence in overall ratings; Very Low = Very low level of confidence in overall ratings; For the hypothesis testing on difference between subgroups, Cohen's d was calculated using the formulas presented by Friedman (1968), and Thalheimer and Cook (2002).
